# Musculoskeletal adverse events in dogs receiving bedinvetmab (Librela)

**DOI:** 10.3389/fvets.2025.1581490

**Published:** 2025-05-09

**Authors:** Mike Farrell, Felix W. A. Waibel, Ines Carrera, Giliola Spattini, Louise Clark, Robert J. Adams, Dirsko J. F. Von Pfeil, Ricardo J. R. De Sousa, Diego Bobis Villagrà, Maria Amengual-Vila, Annalisa Paviotti, Rob Quinn, Justin Harper, Stephen P. Clarke, Christopher J. Jordan, Michael Hamilton, Andy P. Moores, Mark Irwin Greene

**Affiliations:** ^1^Ferguson Veterinary Clinic, Glenrothes, United Kingdom; ^2^Department of Orthopaedic Surgery, Balgrist University Hospital, Zurich, Switzerland; ^3^Vetoracle Teleradiology, Diss, United Kingdom; ^4^Clinica Veterinaria Castellarano, Castellarano, Italy; ^5^Davies Veterinary Specialists, Shillington, United Kingdom; ^6^Paragon Veterinary Referrals, Wakefield, United Kingdom; ^7^Small Animal Surgery Locum, PLLC, Dallas, TX, United States; ^8^ESPEVET, Mallorca, Spain; ^9^Vets Now Limited, Dunfermline, United Kingdom; ^10^Frank Pet Surgeons, Leeds, United Kingdom; ^11^Vita Referrals, Wetherby, United Kingdom; ^12^Texas Specialty Veterinary Services, Boerne, TX, United States; ^13^The Moores Orthopaedic Clinic, Basingstoke, United Kingdom; ^14^Hamilton Specialist Referrals, High Wycombe, United Kingdom; ^15^Perelman School of Medicine, University of Pennsylvania, Philadelphia, PA, United States

**Keywords:** bedinvetmab, Librela, NGF, rapidly progressive osteoarthritis, RPOA, accelerated joint destruction

## Abstract

**Objectives:**

To conduct a specialist-led disproportionality analysis of musculoskeletal adverse event reports (MSAERs) in dogs treated with bedinvetmab (Librela™) compared to six comparator drugs with the same indication. Furthermore, to report the findings from a subset of dogs whose adverse event (AE) data underwent independent adjudication by an expert panel.

**Study design:**

Case–control study and case series analysis.

**Sample population:**

The European Medicines Agency’s EudraVigilance database (2004–2024) and 19 client-owned dogs.

**Methods:**

An EBVS^®^ Veterinary Specialist in Surgery individually reviewed all MSAERs to Librela™, Rimadyl^®^, Metacam^®^, Previcox^®^, Onsior^®^, Galliprant^®^, and Daxocox^®^ (2004–2024). The primary null hypothesis was that Librela’s MSAER rate would not exceed that of comparator drugs by more than 50%. The secondary hypothesis was that MSAER would surge and taper following the launch of new drugs.

**Results:**

The disproportionality analysis did not support the hypotheses. Ligament/tendon injury, polyarthritis, fracture, musculoskeletal neoplasia, and septic arthritis were reported ~9-times more frequently in Librela-treated dogs than the combined total of dogs treated with the comparator drugs. A review of 19 suspected musculoskeletal adverse events (MSAEs) by an 18-member expert panel unanimously concluded a strong suspicion of a causal association between bedinvetmab and accelerated joint destruction.

**Conclusion:**

This study supports recent FDA analyses by demonstrating an increased reporting rate of musculoskeletal adverse events in dogs treated with Librela. Further investigation and close clinical monitoring of treated dogs are warranted.

**Impact:**

Our findings should serve as a catalyst for large-scale investigations into bedinvetmab’s risks and pharmacovigilance.

## 1 Introduction

Osteoarthritis (OA) is the most prevalent chronic pain condition in companion animals, and is a significant contributor to reduced quality of life and premature death (1). Although a diverse array of therapeutic approaches are currently available, all possess limitations, including suboptimal efficacy and the potential for severe adverse reactions. Chronic pain management is challenged by the trade-off between safety and efficacy. Analgesic drugs that provide significant pain relief can carry a higher risk of adverse reactions than safer, less effective options like glucosamine-chondroitin joint supplements (2). This therapeutic dilemma is complicated by differing risk perceptions between veterinarians and caregivers. Veterinarians, due to their medical training, may be more comfortable with the risks associated with prescription analgesics, whereas caregivers may be more hesitant, sometimes declining or limiting their use even when deemed necessary by the veterinarian (3). This disconnect is underscored by a 2018 study revealing that 22% of dogs for whom analgesics were recommended by their veterinarians did not receive them (4).

Development of new therapies offering enhanced safety and efficacy can help bridge the gap between veterinary recommendations and caregiver acceptance. Bedinvetmab (Librela™), a monoclonal antibody (mAb) targeting nerve growth factor (NGF), represents a significant advancement in canine osteoarthritis (OA) pain management. Following its approval by the European Commission in November 2020, it became the first mAb authorized for this indication. The Food and Drug Administration (FDA) granted U.S. marketing authorization 2.5 years later, and the Australian Pesticides and Veterinary Medicines Authority subsequently registered the same drug as Beransa™.

While these regulatory approvals underscored worldwide confidence in veterinary mAbs, their human equivalent were associated with substantial safety concerns. Specifically, NGF modulates bone and cartilage turnover (5), and its inhibition is linked to accelerated joint degeneration in humans (6, 7). This was evidenced in 2012, when clinical trials of anti-NGF mAbs (aNGFmAbs) revealed rapidly progressive osteoarthritis (RPOA) (8), leading the FDA to impose a two-year clinical trial hold and mandate a risk evaluation and mitigation strategy (REMS) post-hold. However, even with stringent screening, low dosing, and NSAID prohibition after the REMS was implemented, RPOA risk persisted (9–11). While the exact mechanism is still under investigation, human clinical trials did not support the hypothesis that RPOA is caused by overuse of weight-bearing joints (7, 10).

Post-marketing pharmacovigilance is crucial for continuously monitoring a drug’s safety and efficacy after it enters the market, as clinical trials cannot capture the full spectrum of potential adverse reactions. It involves a combination of voluntary reporting of adverse drug reactions (ADRs) by healthcare professionals and the public, and proactive surveillance programs, including government-funded, industry-sponsored, and independent research. Their findings inform regulatory decisions, which can range from label updates and limited use restrictions to, in rare cases, market withdrawal if the risks outweigh the benefits (12).

When an animal exhibits unexpected clinical signs following drug administration, differentiating these effects from the underlying disease or a new unrelated condition can be complex. Nevertheless, prompt identification of potential causal relationships is paramount for ensuring patient safety. The thalidomide tragedy exemplifies the critical importance of rigorous pre-clinical and post-marketing drug safety surveillance. Insufficient testing and a lack of robust post-marketing surveillance systems failed to identify the teratogenic potential of thalidomide (13). This resulted in widespread use of the drug leading to severe birth defects in thousands of children, highlighting the potentially devastating consequences of delayed recognition of drug-related adverse events.

In December 2024, the FDA issued an open letter to veterinarians, alerting them to neurological and musculoskeletal safety signals identified during their post-marketing surveillance of Librela (14). The FDA’s Center for Veterinary Medicine (CVM) employed an algorithmic approach to evaluate ADRs. Their approach, incorporating disproportionality analysis, statistically assessed the frequency of reported adverse events (AEs) in dogs receiving Librela compared to those treated with other OA medications. The FDA’s analysis identified 18 distinct safety signals in dogs administered Librela, encompassing neurological events, urinary problems, and musculoskeletal disorders (14). Notably, the FDA observed a disproportionately elevated reporting rate of “lameness” in dogs receiving Librela. In response, the Center for Veterinary Medicine (CVM) advised veterinarians to proactively inform pet owners about these potential adverse reactions (14).

The CVM emphasized that their objective was to generate hypotheses, acknowledging the inherent limitations of establishing definitive causality (14). They noted that AE reporting systems are subject to various biases, including underreporting, reporting biases influenced by [social] media attention, and confounding factors such as concomitant medications. Furthermore, the CVM’s reliance on algorithmic analyses of secondary data, without the benefit of expert clinical interpretation, introduces additional diagnostic uncertainty (15). To address this limitation, we employed a two-pronged approach. Firstly, we conducted a specialist-led disproportionality analysis of musculoskeletal adverse event reports (MSAERs) to expand upon the CVM’s work. This analysis tested the null hypothesis that Librela’s MSAER rate would not exceed that of six comparator drugs with the same indication by more than 50%. Secondly, we report the findings from a subset of dogs whose AE data were subjected to independent adjudication by an expert panel and subsequently submitted to the European Medicines Agency (EMA).

## 2 Materials and methods

### 2.1 MSAER disproportionality analysis

**Figure 1:**
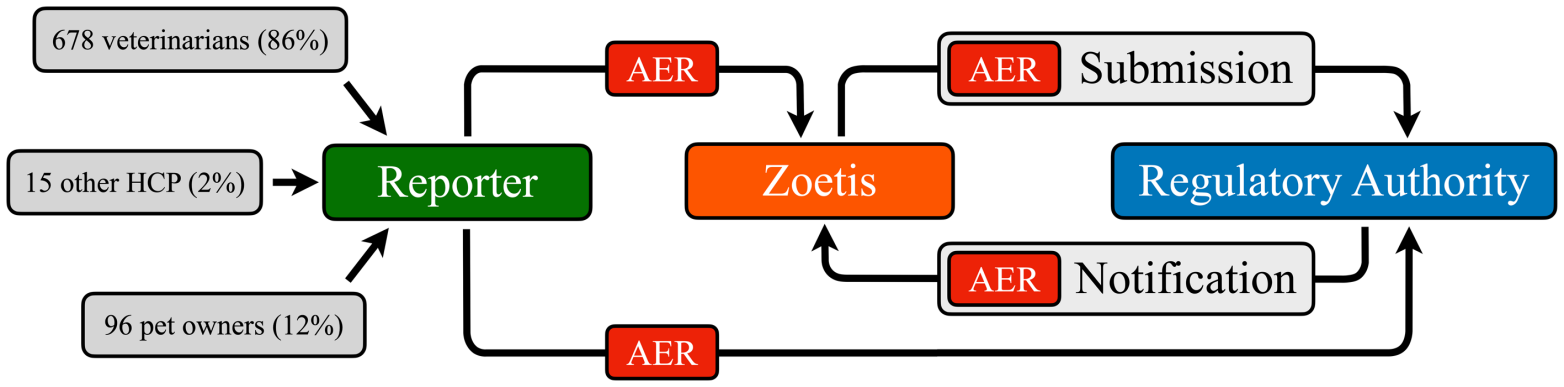
This diagram is adapted from the European Medicines Agency’s pharmacovigilance data flow chart (36). Although the Agency encourages reporting via the attending veterinarian (shown in the upper half of the diagram), an Adverse Event Report (AER) may be initiated by anyone directly involved with the event. Crucially, this system lacks a direct feedback loop to the first-hand reporter. A lack of feedback hinders transparency, increases the risk of errors, and has the potential to discourage future reporting. Note that 88% of AERs were submitted by veterinarians or other healthcare professionals (HCPs), a finding that does not support Zoetis’ concerns regarding potential over-reporting influenced by negative social media activity directed at Librela (22).

A detailed description of the EudraVigilance database (EVD) analysis is provided in Supplementary Video 1. Briefly, accurate identification of clinically relevant musculoskeletal adverse events (MSAEs) required a thorough understanding of the pharmacovigilance system’s information flow (Figure 1), and the limitations inherent in the system. Specifically, diagnostic terms submitted by primary reporters are only published if they are listed in the Veterinary Dictionary for Drug Regulatory Activities (VeDDRA). Otherwise, the Marketing Authorization Holder (MAH; Zoetis, Louvain-la-Neuve, Belgium) selects a diagnostic term from a predefined list including clinical signs (e.g., lameness), non-specific diagnoses (e.g., arthritis), and specific diagnoses (e.g., ligament rupture) (16). At the time of analysis, VeDDRA contained 113 musculoskeletal and 313 neurological AE diagnoses (i.e., low-level terms, LLTs) (16). Many LLTs exhibit clinically relevant overlap (Table 1). For example, “limb weakness” (a musculoskeletal LLT) may indicate a neurological problem, while “collapse of leg” (a neurological LLT) might describe an orthopedic AE. With over 35,000 possible combinations of musculoskeletal and neurological LLTs, a simple algorithmic approach was not considered feasible.

**Table 1:** All AE diagnoses in the EVD must be low-level terms (LLTs) published in the Veterinary Dictionary for Drug Regulatory Activities (VeDDRA) (16).

	Musculoskeletal	Neurological	Systemic
Functional problem	Lameness	Walking difficulty	General pain
	Difficulty to rise	Unable to stand	Reluctant to move
	Difficulty going up/down stairs	Collapse of leg	Recumbency
Physical exam finding	Joint effusion	Muscle atrophy	Swollen limb
Non-specific diagnosis	Arthritis		
	Arthrosis		
	Bone and joint disorder		
	Joint cartilage disorder		
	Luxation/subluxation		
	Fracture		
Total number of LLT	113	313	322

Accurate identification of clinically relevant MSAEs presented significant challenges due to ambiguous diagnostic terms and potentially relevant overlap between musculoskeletal and neurological AEs. The challenge was compounded by concurrent systemic AEs: For instance, a dog with “arthrosis” and “acute renal failure” resulting in death would be excluded. Ultimately, 79.5% of AERs were deemed ineligible as primary musculoskeletal problems (Supplementary Video 1).

To ensure consistency and account for the complex clinical judgments required for data interpretation, a single EBVS^®^ Specialist in Surgery (Author 1) reviewed musculoskeletal adverse event reports (MSAERs) logged within the European Medicines Agency’s (EMA) EudraVigilance database (EVD) from its 2004 inception to December 31, 2024. A descriptive disproportionality analysis was used to compare the incidence of MSAERs associated with Librela to those of six other veterinary analgesics: Rimadyl^®^, Metacam^®^, Previcox^®^, Onsior^®^, Galliprant^®^, and Daxocox^®^. This analysis aimed to identify any potential temporal trends in MSAER reporting, particularly following the introduction of new medications. All adverse event reports (AERs) are filed in the EVD under trade names; therefore, trade names are used consistently throughout this manuscript.

### 2.2 Case series inclusion criteria

This study utilized a retrospective, case series design. Case recruitment was initiated by Author 1 following the observation of a suspected case of RPOA in a dog receiving Librela. This case was shared on a specialist veterinary forum (17). Subsequently, over an 11-month period, multiple clinicians who subscribed to the forum contacted Author 1 to share concerns regarding serious MSAEs in dogs treated with Librela. Based on these communications, an independent working group was formed comprising clinicians with firsthand experience with MSAEs (see below). The primary objective was to investigate a potential association between Librela administration and the observed pathology. Given the lack of prior records of RPOA in dogs, the working group sought advice from an expert in human neuro-osteoarthropathy (Author 2) and two EBVS^®^ Specialists in Diagnostic Imaging with published expertise in musculoskeletal imaging (Authors 3 and 4).

Twenty-three suspected musculoskeletal adverse events (MSAEs) were independently reviewed by nine investigators with a combined 128 years of experience in referral practice (Authors 1, 5, 6, 7, 9, 11, 14, 15, 16). Clinical data from each case, including signalment, clinical signs, Librela dosing information, concurrent medications, treatment outcomes, and relevant diagnostic test results (radiographs, CT scans, synovial fluid analysis, histopathology), were evaluated. Four cases were excluded from further analysis due to incomplete data or insufficient evidence to support a causal relationship. Twelve MSAERs had already been filed in the EVD, and retrospective reports were filed for the remaining cases at this time.

### 2.3 Case series adjudication

The independent adjudication panel comprised 12 veterinary orthopedic surgeons, an orthopedic consultant specializing in human neuro-osteoarthropathy, two veterinary diagnostic imaging specialists, two veterinary anesthetists, and a cancer researcher with expertise in monoclonal receptor-based therapeutics. The adjudication panel demonstrated collective expertise including 157 relevant peer-reviewed publications spanning monoclonal antibodies, neuropathic arthropathy, canine osteoarthritis (OA), pathological fractures, and humeral intracondylar fissure (HIF).

Transcripts from the 2012 and 2021 Arthritis Advisory Committee (AAC) and Drug Safety and Risk Management Advisory Committee Meetings were reviewed. Our analysis focused on their joint safety review of humanized anti-nerve growth factor monoclonal antibodies (aNGFmAbs) (8–10). The following limitations in clinical trials used to define human RPOA were acknowledged:

1. *Inconsistent baseline imaging:* Humans enrolled in low-back pain aNGFmAb clinical trials who developed RPOA did not undergo baseline radiographic imaging of the affected joint (s) before starting treatment (8, 18).
2. *Nonspecific terminology:* The definition of human RPOA included joint pathology “falling well outside the natural history of OA” (8). Notably, this criterion lacked a specific definition of the “natural history of OA” and did not reference a control group with typical OA progression.
3. *Inapplicability of the human definition:* The specific term “RPOA” was not adopted for our case adjudication due to its reliance on measurements of large human joints using standing radiographs or MRI (11).

Nineteen dogs with suspected MSAEs following bedinvetmab treatment were independently evaluated by Authors 3 and 4. Suspected drug-related AEs were defined according to the AAC’s methodology as joint pathology “falling well outside the natural history of OA” (8). This included pathological fractures or luxations in osteoarthritic joints, and subchondral osteolysis in the absence of clinical evidence of septic or immune-mediated arthritis. Inter-rater agreement for the two diagnostic imaging specialists was tested using the Fleiss *κ* coefficient (19).

Diagnostic images were formatted and annotated by Authors 1 and 3. Subsequently, all 18 experts independently reviewed the annotated images, emulating standard clinical practice. The entire cohort of 19 cases was evaluated collectively by the adjudication panel to determine potential drug causality, rather than assessing each case individually, mirroring the AAC’s 2012 protocol (8). Readers can review the cases by watching Supplementary Video 2.

A three-tiered system was used to describe a potential causal relationship between bedinvetmab treatment and MSAEs. Outcomes explicitly implying a known causal link (e.g., “definitely related”) were avoided to reflect the inherent uncertainty of this assessment. Instead, the experts described their personal judgment as “very suspicious,” “suspicious,” or “insufficient evidence” of a potential causal relationship.

### 2.4 AER translation errors

Translation errors were identified by comparing MSAERs submitted by attending veterinarians with corresponding reports filed by the MAH. A clinically relevant discrepancy between the reports was considered a translation error.

## 3 Results

### 3.1 MSAER disproportionality analysis

A total of 4,746 MSAERs were identified between May 20, 2021 (3 months after Librela’s European release) and December 31, 2024. Following the exclusion of 457 comparator medication reports which specified co-administration of Librela, 4,289 MSAERs remained. Of these, 3,755 (87.5%) were attributed to Librela. The majority of MSAERs (3,411, 79.5%) were excluded due to confounding neurological and/or systemic/neoplastic diagnoses (Supplementary Video 1), resulting in a final cohort of 878 eligible MSAERs for analysis, with 789 (90%) attributed to Librela (Supplementary Table S1). Most primary reports to Librela (88%) were submitted by veterinarians and other healthcare professionals (Figure 1).

**Figure 2:**
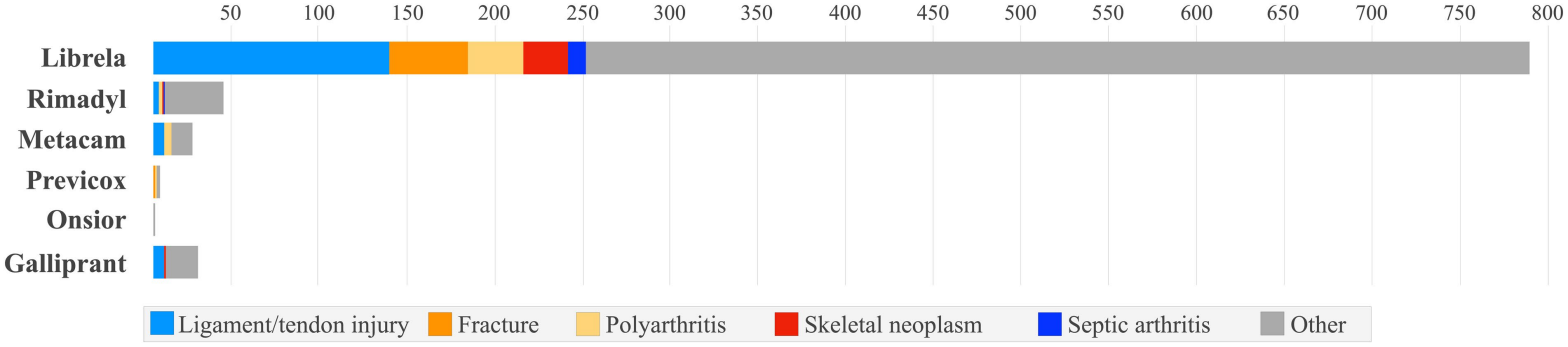
Accumulated MSAER comparing Librela to six medications with the same indication, focusing on specific reaction types. Ligament/tendon injury, polyarthritis, fracture, musculoskeletal neoplasia, and septic arthritis were reported ~9 times more frequently in dogs treated with Librela than the combined total of dogs treated with the comparator medications. No qualifying MSAER were found for Daxocox^®^ (enflicoxib), a weekly NSAID released in April 2021, 2 months after Librela’s European launch.

**Figure 3:**
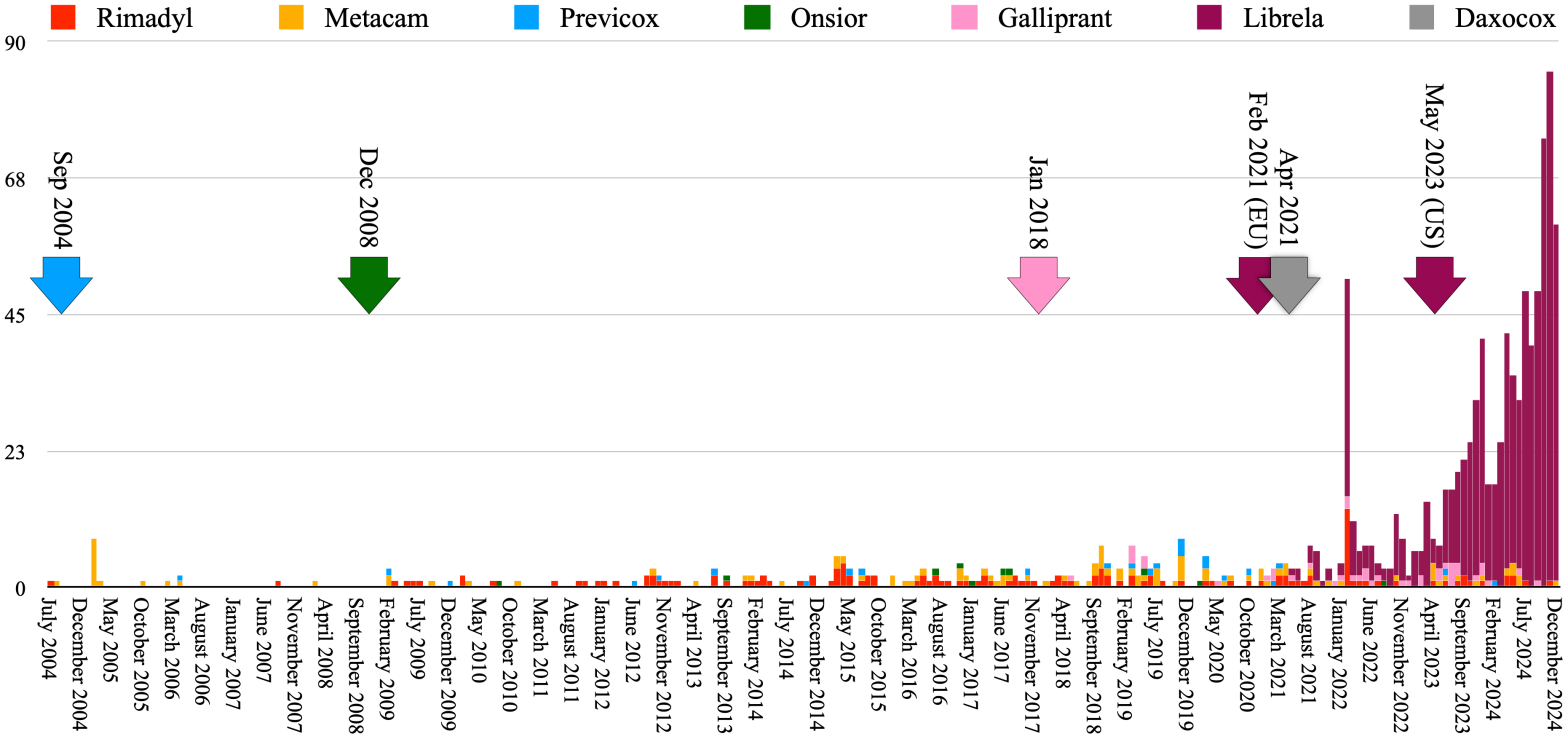
Accumulated MSAER for Librela and the same comparator medications as shown in Figure 2. There was a dramatic increase in MSAER to Librela in the second half of 2024, reaching ~4-times the level observed in the 6-months following its 2023 U.S. release and ~59-times the level observed in the 6-months following its 2021 European release. To test the hypothesis that MSAER would surge and taper following the launch of new drugs (indicated by color-coded arrows), we analyzed data from the EudraVigilance database since its 2004 inception. Neither this hypothesis nor the primary null hypothesis—that Librela’s MSAER would not exceed those of comparator drugs by more than 50%—were supported by the data. In fact, Librela’s accumulated MSAER measured over 45 months were ~3-times higher than the combined accumulated MSAER of all comparator drugs measured over 240 months. The February 2022 MSAER spike might reflect the EMA’s mandate for MAHs to submit all previously un-submitted AERs at regular intervals (43). Post-release MSAER trends for Rimadyl^®^ and Metacam^®^ are unavailable because they were released in 1997 and 1998, respectively.

Ligament/tendon injury, polyarthritis, fracture, musculoskeletal neoplasia, and septic arthritis were reported ~9-times more frequently in Librela-treated dogs than the combined total of dogs treated with the comparator medications (Figure 2). Furthermore, accumulated MSAERs for Librela over 45 months exceeded those of the highest-ranking NSAID (Rimadyl) by ~20-fold and surpassed the combined accumulated MSAERs of all comparator drugs over 240 months by ~3-fold (Figure 3). These findings did not support the null hypothesis that Librela’s MSAER rate would not exceed that of comparator drugs by more than 50%. Moreover, the secondary hypothesis that MSAER would surge and taper following the launch of new drugs was not supported (Figure 3).

### 3.2 Specific diagnoses and outcomes for Librela’s MSAERs

**Figure 4:**
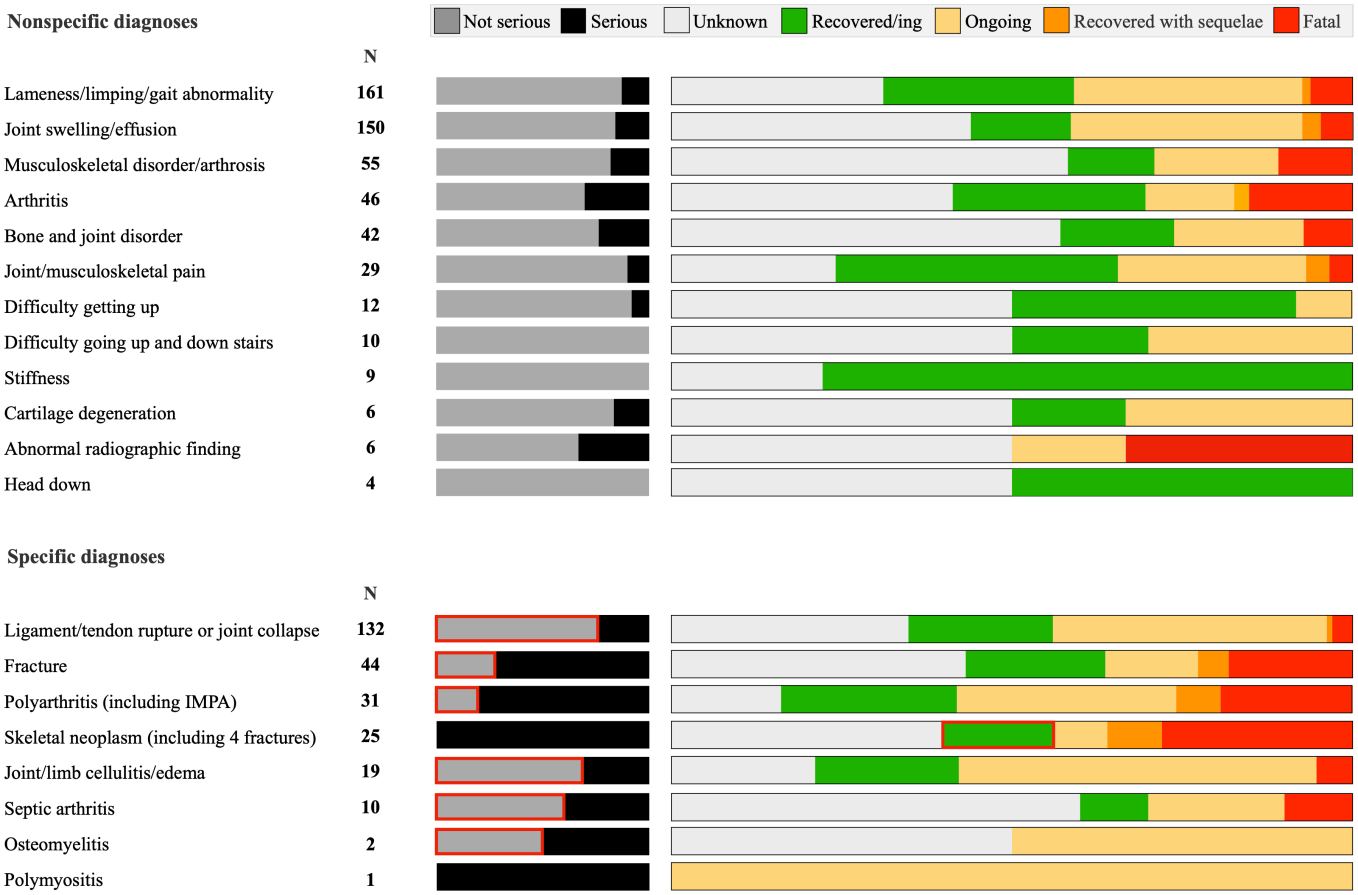
Severity and outcome data for MSAER associated with Librela. Unexpected findings are highlighted in red. These include a significant proportion of severe MSAER, such as ligament ruptures, luxations, fractures, limb collapse, and septic arthritis, filed as “not serious”. In addition, several dogs diagnosed with bone cancer were reported as “recovered/resolving”. The EMA defines a serious adverse event as “any adverse event which results in death, is life-threatening, results in persistent or significant disability/incapacity, or a congenital anomaly or birth defect.” However, a more intuitive and clinically relevant definition includes events causing permanent disability (44), requiring surgical intervention and/or prolonged hospitalization (12). Importantly, published AER data are subject to change, but only if translation errors are recognized and reported (see Figure 1).

The most frequent diagnostic terms selected by the MAH were “arthritis” or associated clinical signs (e.g., “lameness”, “joint pain”, “difficulty climbing stairs”), encompassing 530 cases (67%) (Figure 4). Of these, the MAH filed 442 reports (83.4%) as “not serious”. The remaining 259 ADRs included ligament injuries, limb collapse, polyarthritis, bone cancer, and fractures. Among these, the MAH filed 138 reports (52.3%) as “not serious”.

The most frequently reported outcome was “unknown” (310 dogs; 39%). Of the remaining dogs, 177 (22%) experienced AEs that were reported as “recovered/resolving/normal”; 229 (29%) were filed as “ongoing”; 15 (2%) “recovered with sequelae”; and 63 dogs (8%) died or had been euthanized.

### 3.3 Case series adjudication outcome

**Figure 5:**
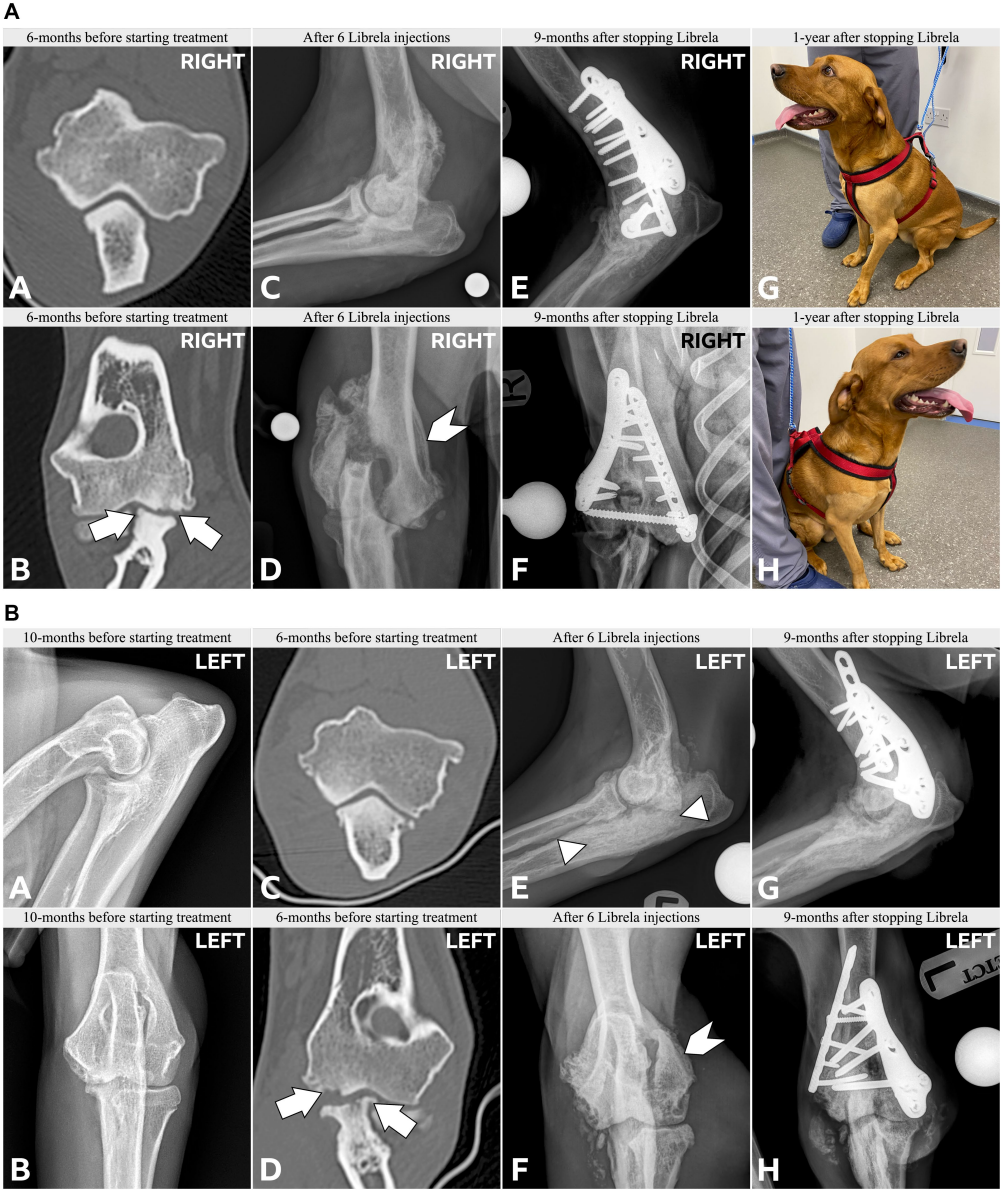
**(A)** Case 1, a young adult Labrador Retriever. A,B: Orthogonal CT slices demonstrating medial compartment pathology within the right elbow joint. Arrows indicate a medial coronoid process (MCP) fissure and humeral condylar kissing lesion. There is no evidence of a humeral intracondylar fissure (HIF). C,D: Preoperative radiographs obtained after 6 Librela injections reveal a lateral humeral condylar fracture (HCF) with subsequent malunion. A chevron symbol highlights atypical periosteal new bone growth along the medial epicondyle [see Panel 5B(F)]. E,F: Postoperative radiographs acquired 9 months after surgery show successful fracture union, although subchondral bone resorption had progressed. G,H: Severe bilateral seromas that developed after two Librela injections and persisted despite treatment. **(B)** Case 1—contralateral elbow. A–D: Pre-treatment radiographs and CT scans show MCP remodeling and a humeral condylar lesion consistent with osteochondrosis. E,F: Severe proximal ulnar sclerosis and atypical medial epicondylar periosteal new bone formation were observed after 6 doses of Librela. G,H: Surgical intervention was performed 3 months later. Surgery resulted in successful fracture union, but persistent clinical dysfunction necessitated euthanasia. Histopathological examination: Surgical site infection and septic arthritis were excluded. Findings were consistent with those described in an article on RPOA submitted to the pathologist by the attending specialist (20). AER: The MAH filed a report with an incorrect diagnosis of septic arthritis (Supplementary Figure S1).

**Figure 6:**
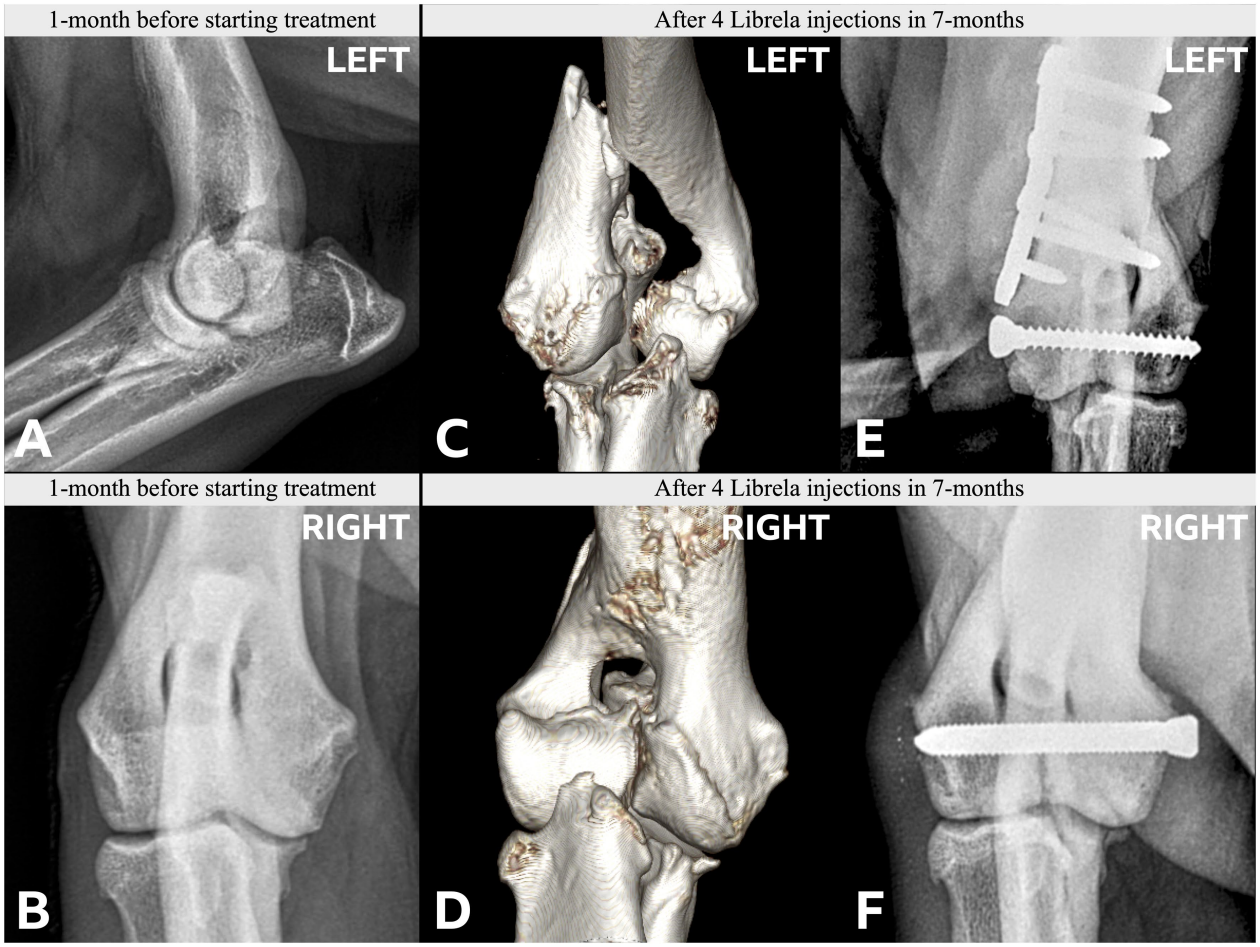
Case 4 is a Labrador Retriever with no history of trauma. **(A,B)** Pre-treatment radiographs taken aged 8.5-years. A pre-treatment left craniocaudal radiograph was not acquired. **(C,D)** Post-Librela radiographs demonstrate a left lateral HCF with an atypical medial epicondylar periosteal reaction **(C)** and a right HIF **(D)**. **(E,F)** Immediate post-operative radiographs. AER: After considering the lack of trauma and atypical signalment for HIF, the attending specialist filed an AER to the Veterinary Medicines Directorate (VMD) for suspected RPOA. This report was shared with the MAH, who filed a report for non-serious “arthritis”, with an outcome of recovered/resolving (Supplementary Figure S3).

**Figure 7:**
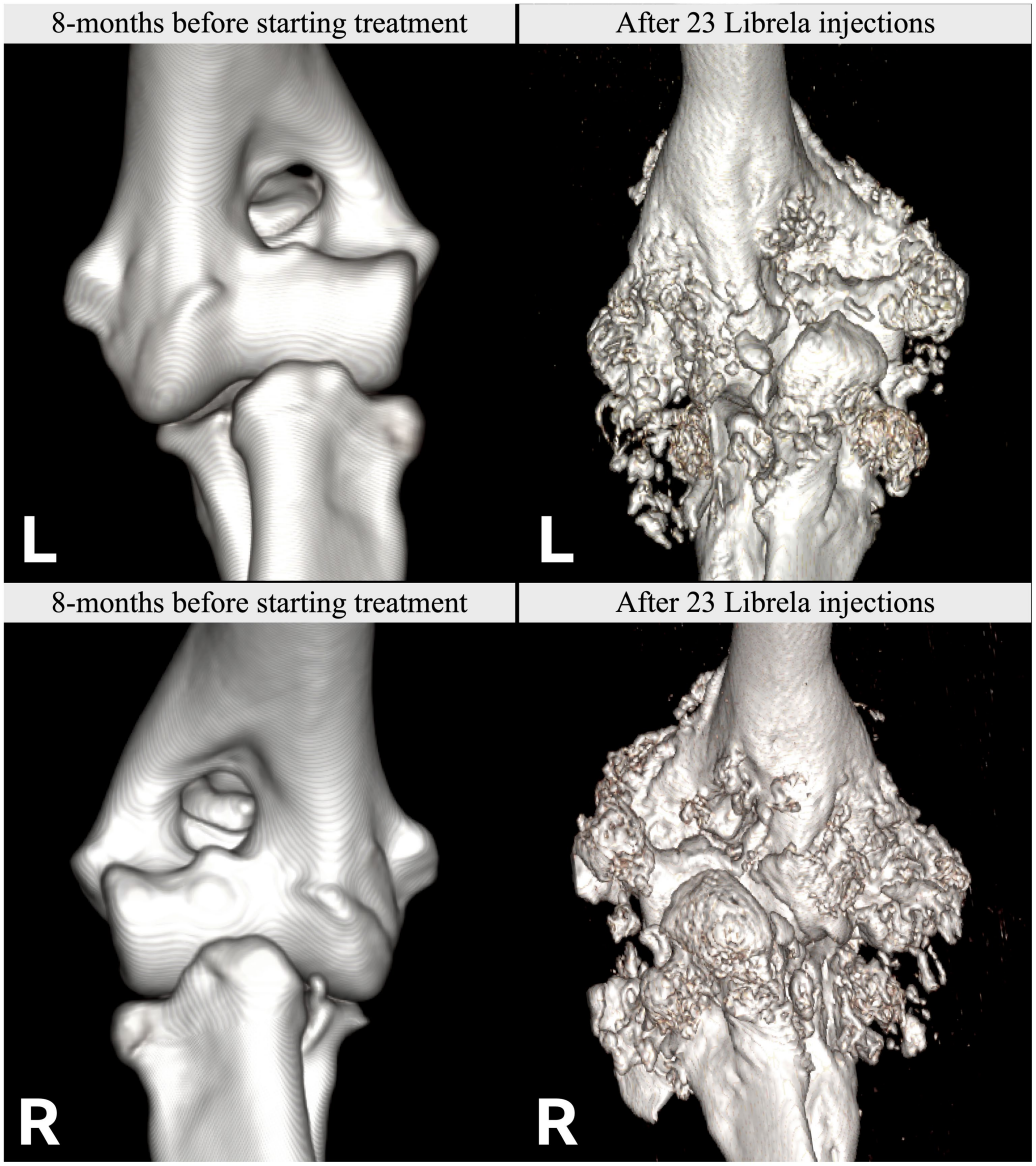
Left (L): Normal pre-treatment CT scans from Case 11, a 7.5-year-old Labrador Retriever. Post-Librela CT scans revealing fulminant periarticular osteophytosis. Right (R): Post-Librela CT scans revealing fulminant periarticular osteophytosis. AER: The attending specialist filed an AER to the VMD specifying their suspicion of RPOA. This report was shared with the MAH, who filed an AER for non-serious arthritis, with an outcome of recovered/resolving (Supplementary Figure S5).

**Figure 8:**
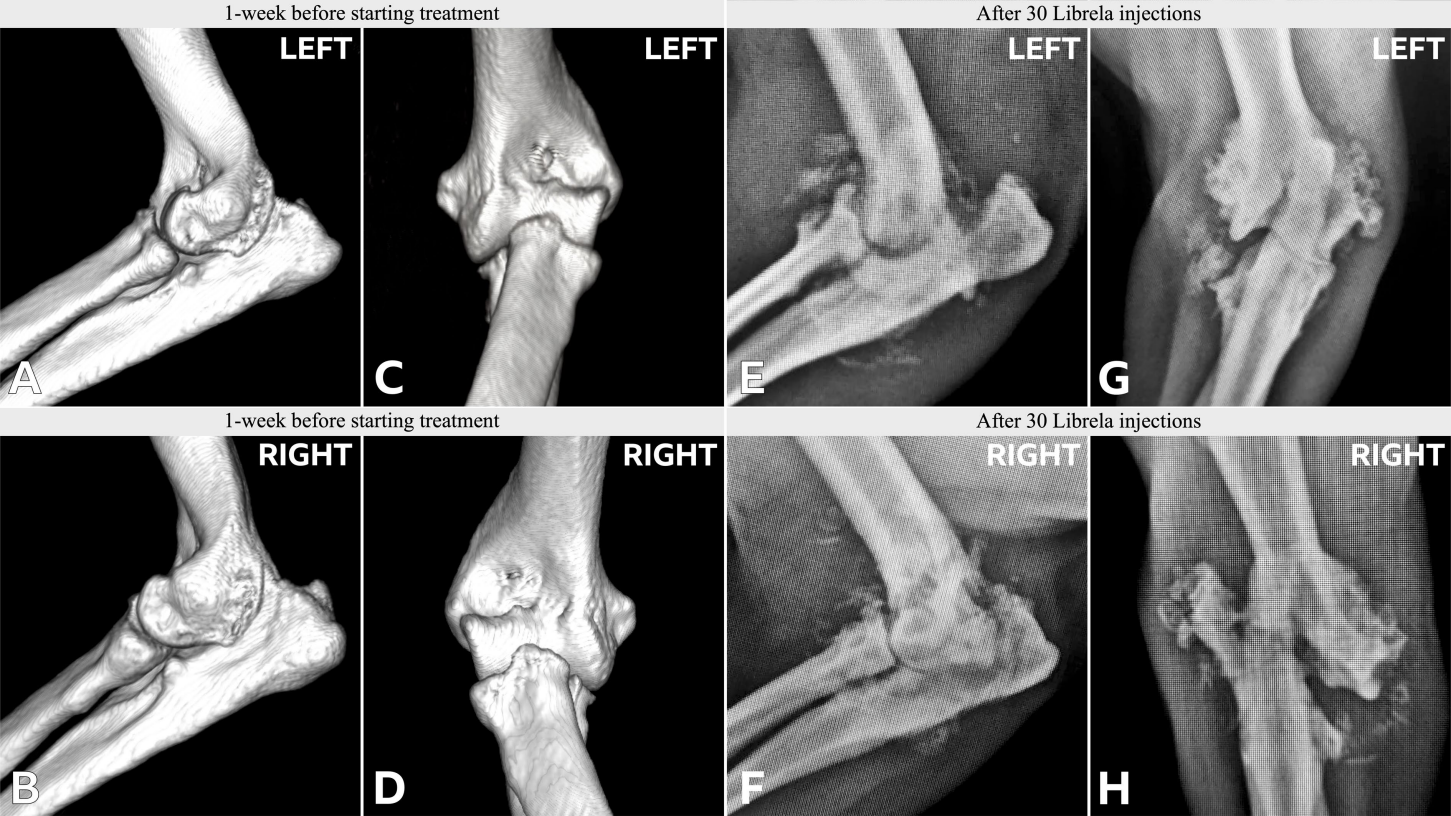
Case 13 is a Staffordshire Bull Terrier with no history of trauma. **(A–D)** Pre-treatment CT scans showed mild elbow arthrosis and excluded HIF. **(E–H)** Post-Librela radiographs demonstrated severe left elbow arthrosis and a right lateral HCF with an atypical medial epicondylar periosteal reaction. AER: Given the patient’s age and breed, which is not predisposed to HCF, the attending specialist submitted a report to the MAH specifying a suspicion of RPOA. The MAH filed a report with an incorrect diagnosis of “osteosarcoma” (Supplementary Figure S7).

**Figure 9:**
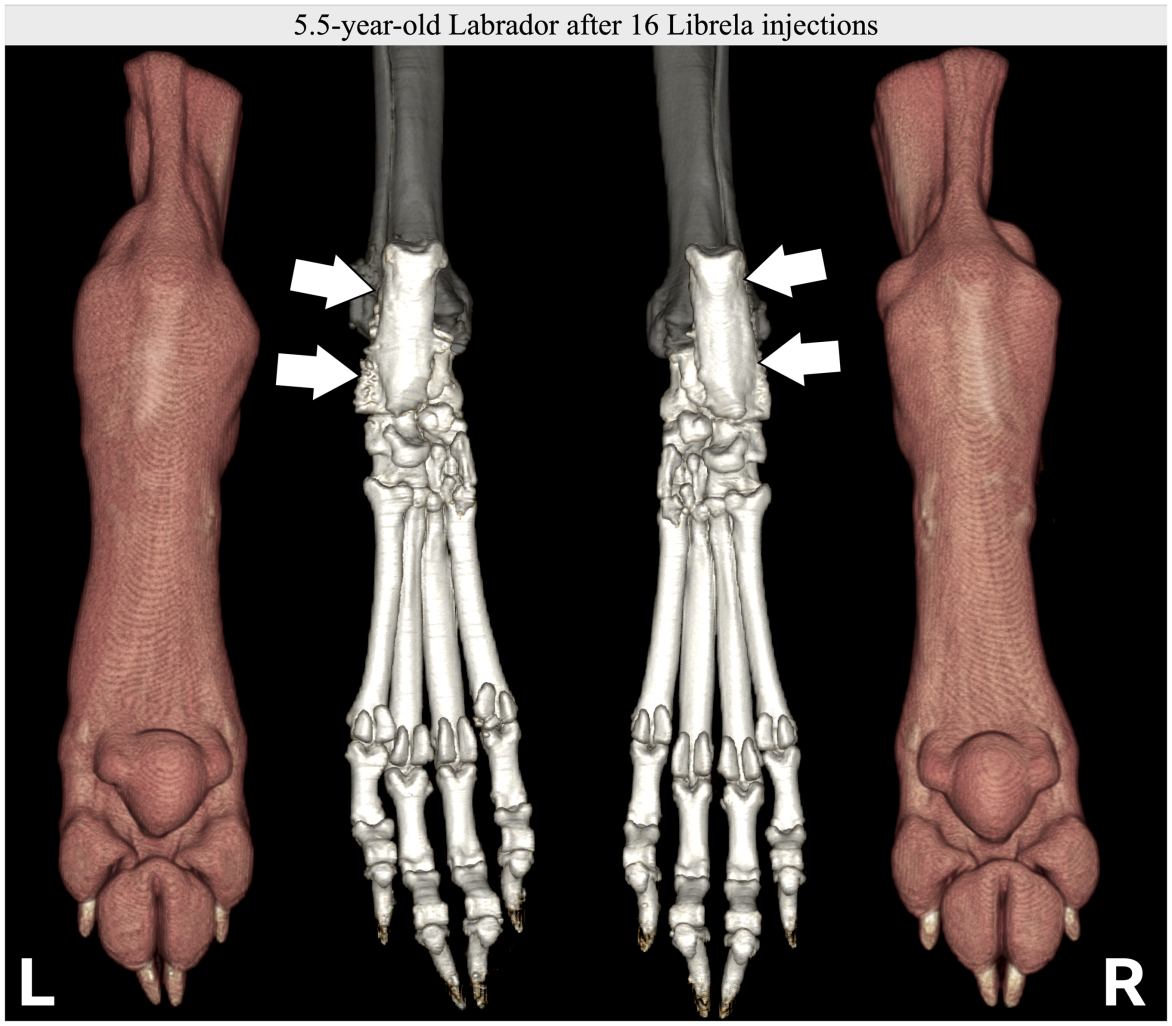
Case 14, a 5.5-year-old Labrador Retriever, received Librela to treat mild elbow dysplasia. Tarsal swelling and hindlimb lameness developed, and this CT scan was acquired after 16 doses. Note the marked bilateral soft tissue swelling and palisading calcaneal periosteal reaction. AER: A report was filed for suspected RPOA because both hocks were clinically normal before treatment and developed severe OA with bilateral talar insufficiency fractures after treatment.

**Figure 10:**
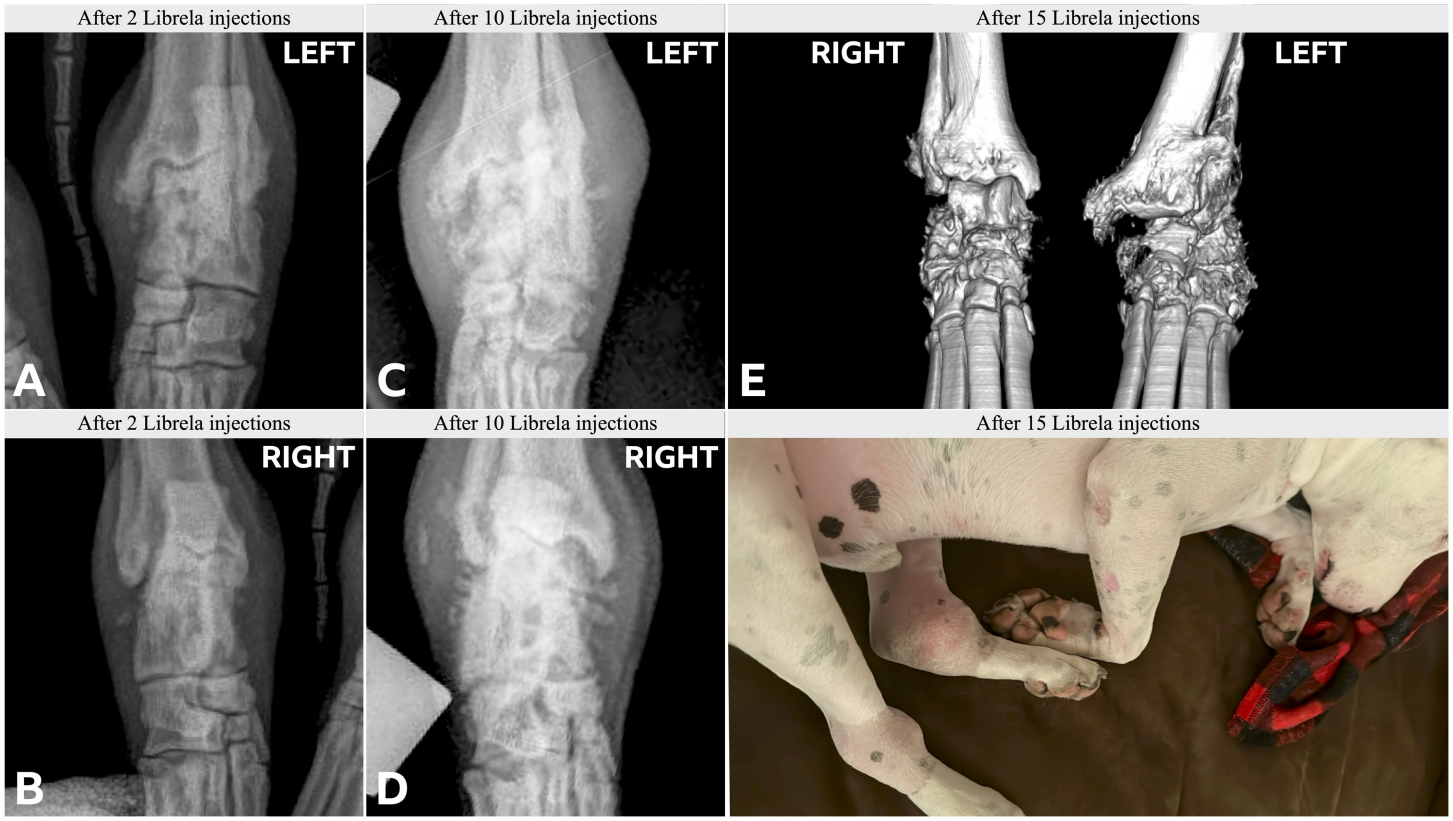
Case 15, an 8-year-old English Bull Terrier treated with Librela for elbow dysplasia developed tarsal swelling and hindlimb lameness within 3 weeks of the first dose. **(A,B)** Radiographs acquired after 2 Librela injections show moderate bilateral arthrosis which progressed rapidly, culminating in left tibiotarsal luxation **(C–E)**. Synovial fluid analysis ruled out inflammatory arthropathy or tick-borne disease. AER: The MAH filed a report using the diagnostic term “swollen joint”, and misclassified the reaction as not serious and recovered/resolving. Following communication from the attending veterinarian regarding the translation error, the MAH escalated the enquiry to their Global Pharmacovigilance Team, who concluded that the observed pathology was consistent with erosive immune-mediated polyarthritis (IMPA), despite normal synovial fluid test results (Supplementary Figure S8).

**Figure 11:**
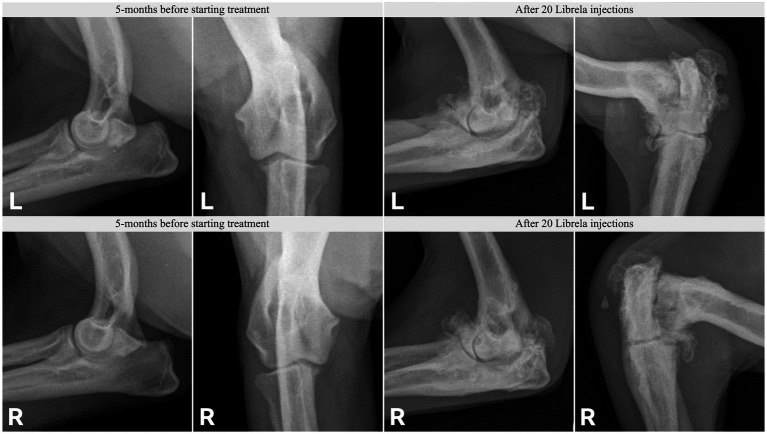
Radiographs from Case 17, a 5-year-old Springer Spaniel, before and after 20 Librela injections.

**Figure 12:**
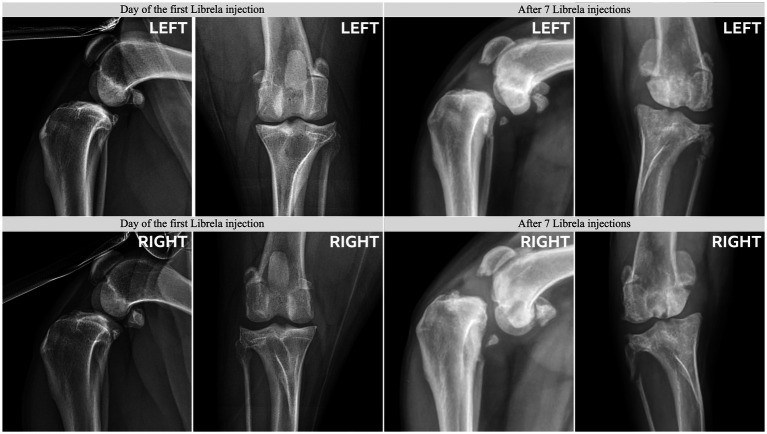
Case 18, a 5.5-year-old Australian Shepherd, had stable stifle joints before starting Librela. AER: The attending specialist filed a report for suspected RPOA to the MAH, who submitted an AER for ligament ruptures, fractures, and joint subluxation/luxations. Their report designated this reaction as not serious (Supplementary Figure S9).

**Figure 13:**
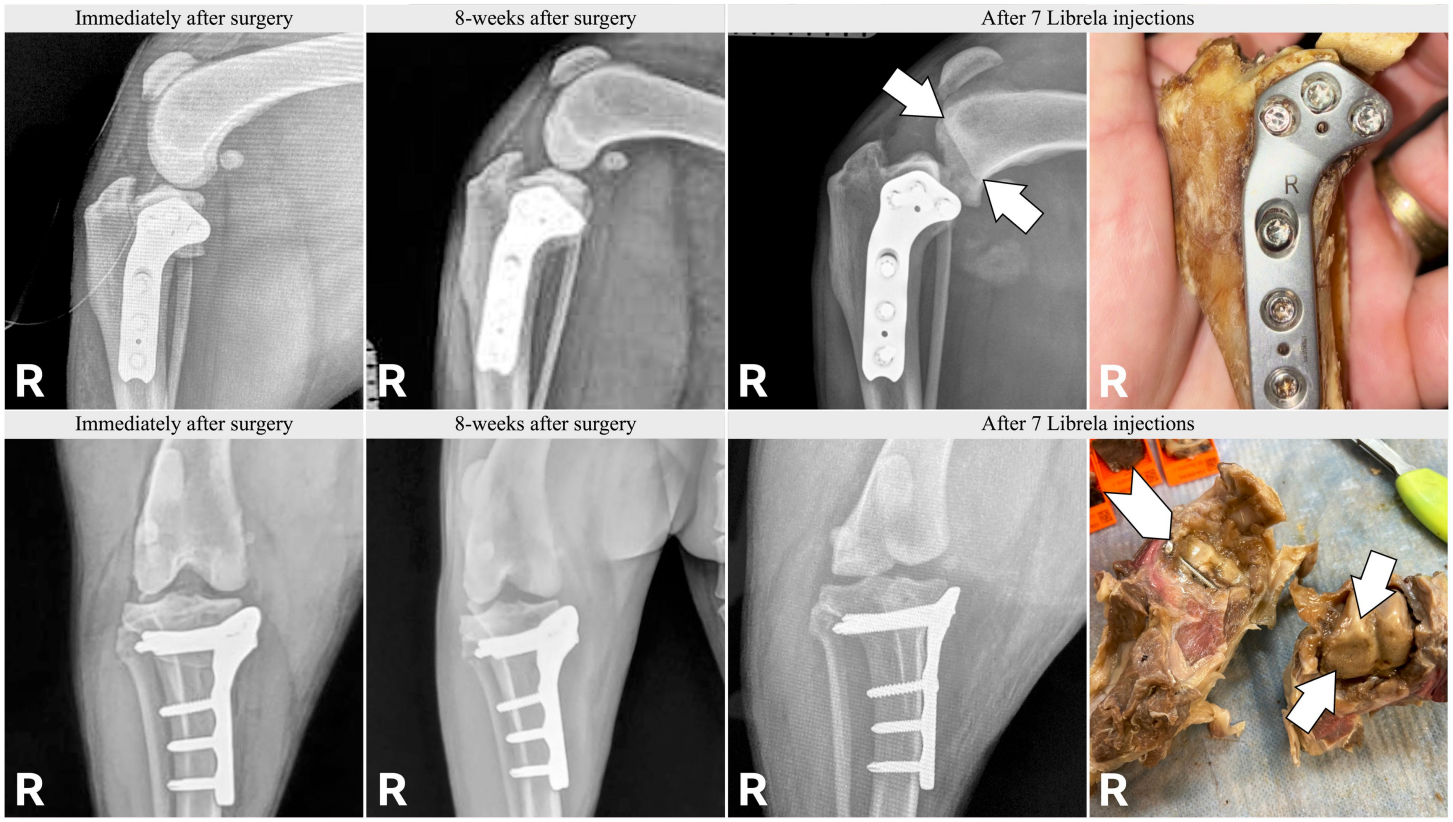
Case 19, a 10.5-year-old mixed-breed dog, had 7 Librela injections following right tibial plateau leveling osteotomy (TPLO). Severe joint erosion is evident in the medial femoral condyle (arrow) and tibial plateau, causing screw exposure (chevron). Intractable pain necessitated euthanasia. Surgical site infection and septic arthritis were excluded by necropsy, which revealed findings similar to those reported in human RPOA (20). AER: The attending specialist filed a report for suspected RPOA to the MAH, who submitted an AER using the diagnostic terms “limb non-weight bearing” and “abnormal radiograph finding”.

Clinical and radiographic characteristics are summarized in Table 2 and Figures 5–13. Mean ± SD number of Librela doses was 12.7 ± 9.5 (range 1–30), with a dose range of 0.4–0.76 mg/kg (mean 0.62 ± 0.08 mg/kg). Referral for investigation of suspected RPOA was made at least 6 months after Librela initiation in 13/19 cases. Eleven dogs (58%) received regular concurrent NSAIDs. The most frequently affected joint was the elbow (13/19 dogs, 68%), followed by the stifle and hock (two dogs each), and hip (one dog). Seven dogs (37%) sustained pathological fractures, and two (10.5%) had joint luxations. Two dogs with clinically normal hock joints before initiating Librela therapy developed severe non-index hock joint destruction after Librela treatment for elbow OA.

**Table 2:** Summary of 19 dogs that experienced joint-related adverse events (MSAEs) while receiving Librela.

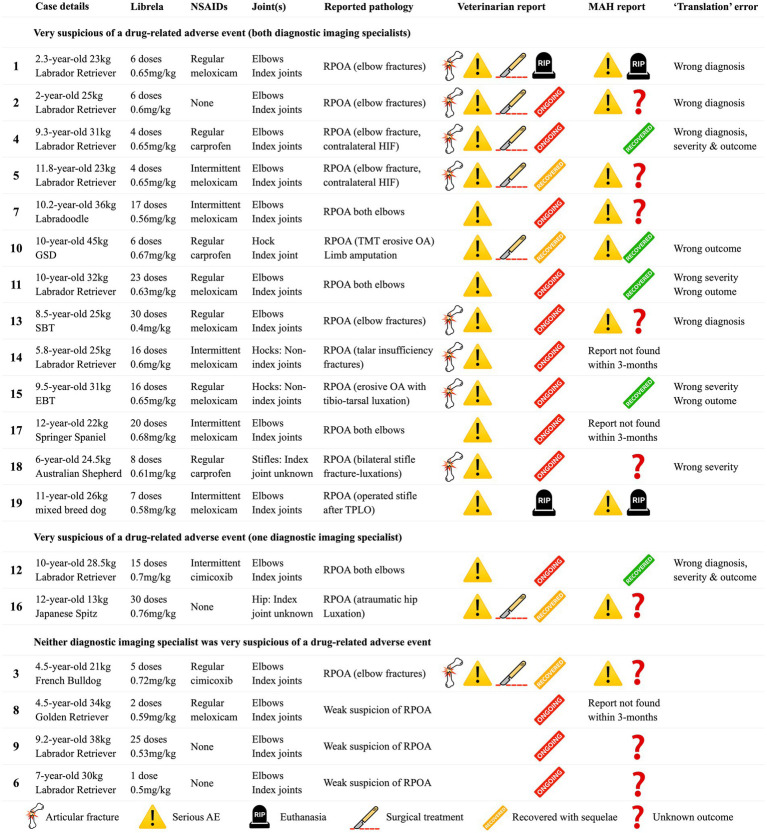

Regular NSAID co-administration was defined as continuous use of NSAIDs for at least 5 days within any 4-week period. Note that some dogs initially received Librela monotherapy and were subsequently started on NSAIDs due to declining treatment efficacy. A “translation error” is defined as a clinically important discrepancy between the AER submitted by the attending veterinarian and the report filed by Zoetis. For example, Case 18, a 6-year-old Australian Shepherd dog experienced bilateral stifle joint luxations and fibular fractures after receiving 8 doses of Librela, and requires sling support for walking. The attending veterinarian reported this as a serious adverse reaction, but the MAH classified it as non-serious (Supplementary Figure S9).

Histopathological examination of bone and synovial tissue from four dogs revealed no evidence of inflammatory arthropathy, tick-borne diseases, or neoplasia. A pathologist who was invited to compare their findings to those reported in a submitted article on human RPOA (20) commented that the pathological features were similar (21).

Interobserver agreement between diagnostic imaging specialists was substantial (*κ* = 0.68, 95% CI 0.4–0.97). Both specialists were very suspicious of a potential causal relationship between the observed pathology and Librela treatment in 68% of dogs (13/19). Furthermore, all 18 panelists (including the two diagnostic imagers) were very suspicious of a potential causal relationship between Librela treatment and the observed pathology.

### 3.4 AER translation errors

Translation errors were identified in 9/19 cases (52%) (Table 2 and Supplementary Figures S1–S6). They included incorrect diagnoses (n = 5), severity (n = 5), and outcome (n = 5). Furthermore, the MAH reported two cases as “overdoses”, despite the administered dosages falling within the recommended range.

## 4 Discussion

This study reveals a striking disparity in musculoskeletal adverse event reports to Librela compared to six comparator drugs. Ligament/tendon injuries, polyarthritis, fractures, musculoskeletal neoplasia, and septic arthritis were reported nine times more frequently in Librela-treated dogs. Worryingly, since its European release, Librela has accumulated 20 times more reports than the highest-ranking comparator drug (Rimadyl) and three times more than *all* comparator drugs combined over a 20-year period. Furthermore, independent expert review of a subset of cases strongly supported a causal association between Librela and accelerated joint destruction.

Librela experienced rapid market penetration following its 2021 European release. Zoetis recently reported global distribution exceeding 21 million doses, translating to an estimated average daily distribution of over 15,000 doses (22). This initial market success has been tempered by emerging concerns regarding bedinvetmab’s safety. These concerns have been amplified by various factors, including the FDA’s safety update (14), negative press coverage (23), the European Commission’s investigation into potential anticompetitive conduct by Zoetis (24), and the emergence of online communities disseminating safety concerns. This confluence of events has fostered a climate of apprehension and confusion. Addressing these concerns requires unbiased and rigorous post-marketing pharmacovigilance to evaluate this drug’s true risk–benefit profile.

Assessing the “expectedness” of adverse drug reactions (ADRs) is fundamental to effective pharmacovigilance. In causal relationship investigations, statisticians use Bayesian principles to evaluate reaction likelihood, considering plausibility and prior knowledge (15). The FDA’s ABON (Algorithm for Bayesian Onset of symptoms) links drug exposure to adverse events AEs (15). For example, when applied to NSAIDs, ABON incorporates prior knowledge of prostaglandin inhibition, its effect on gastrointestinal (GI) mucosal integrity, and the established link between NSAIDs and GI ulceration (25). Notably, NSAIDs can cause subclinical GI damage, undetectable without endoscopy (25). When clinical signs occur, vomiting and diarrhea are common manifestations (26). However, the FDA does not use sales-figure-based prevalence estimates, because they can dramatically underrepresent true incidence (15). For example, comparing carprofen’s AERs to drug sales suggests vomiting and diarrhea occur in <1/10,000 doses, falsely implying that common side-effects are “very rare” (27).

The NSAID analogy is valuable for three reasons. First, while prostaglandins safeguard gastrointestinal integrity, NGF plays a similar pivotal role in bone and cartilage repair (5). Second, serious subclinical cartilage and bone degeneration often precede clinical signs (28). Third, recent claims of bedinvetmab’s “rare” or “very rare” AEs (29) are based on similar methodology to the carprofen analysis described above. Given NGF’s diverse roles and prior evidence of RPOA, subchondral bone fractures, and atraumatic joint luxations in humans and animals (8, 11, 30–33), bedinvetmab-associated MSAEs are an *expected* consequence of NGF inhibition.

Bayesian analysis, while powerful, can be susceptible to subjective biases. This is exemplified by the FDA’s role in the opioid crisis. Despite acknowledging the inherent risk of opioid addiction, the agency over-relied on a five-sentence letter, disproportionately cited as evidence of low addiction rates with oral opioid therapy (34). The FDA’s subsequent mischaracterization of addiction risk as “minimal” was heavily criticized (34). Similarly, the hypothesis that RPOA is a uniquely human problem has faced significant criticism. Multiple experts have contested this claim (32, 35, 36), citing weak supporting data (37). Notably, the joint safety claims outlined in Librela’s datasheet (38) are based on radiographic assessment of five healthy beagles who received the recommended dose (37). This study reported “mild” cartilage erosion in two dogs, despite erosion being, by definition, a severe form of cartilage pathology. Furthermore, despite being invited to provide annotated images to clarify this discrepancy (36), Zoetis declined to do so (39).

Janssen (fulranumab), Pfizer (tanezumab), and Regeneron (fasinumab) self-reported accelerated joint degeneration in their pre-marketing human aNGFmab clinical trials (8). The FDA responded quickly and decisively, voting 21–0 to recognize RPOA as a side-effect of aNGFmAbs and mandating a sophisticated risk mitigation strategy for all subsequent trials (8). The scale of the precautions undertaken by these pharmaceutical companies is exemplified by Pfizer’s tanezumab program, which involved 18,000 patients and 50,000 radiographs analyzed by 250 experts (11).

When viewed in context, bedinvetmab’s limited pre-marketing clinical trials raise serious concerns. Only 89 dogs received more than three doses (40), and crucially, no radiographic screening for accelerated joint degeneration was conducted (40, 41). Unlike Janssen, Pfizer, and Regeneron, Zoetis was unable to self-report accelerated joint destruction due to the absence of radiographic investigations. Consequently, we must rely on post-marketing surveillance to determine whether companion animals experience the adverse joint pathology observed in humans and laboratory animals treated with aNGFmAbs.

We initially intended to publish only the 19 adjudicated cases as a case series. However, we recognized the potential for case examples of severe pathology to be dismissed as outliers—isolated events swamped by the widespread positive experiences reported with bedinvetmab. Given Librela’s popularity, this perspective would be understandable. However, this response would be analogous to assessing the risk of NSAID-induced gastrointestinal harm by comparing the incidence of perforating gastric ulcers with NSAID sales figures. It should be obvious that such an approach neglects the critical fact that NSAIDs can induce subclinical harm which is undetectable without inconvenient tests such as endoscopy. Crucially, unlike the gastrointestinal mucosa, which possesses significant regenerative capacity, cartilage damage, once incurred, is largely irreversible (42). This fundamental difference underscores the gravity of MSAEs associated with aNGFmAbs.

To complement the FDA’s Bayesian analyses, which collected data from May 2023 to March 2024, we employed a descriptive evaluation to 20 years of MSAER data. This approach was deemed complementary because the ABON algorithm primarily focuses on identifying ADRs occurring shortly after medication initiation, while MSAEs often exhibit a long latency period between drug administration and AE detection. This hypothesis is supported by the observation that most reactions in the FDA’s analysis occurred within the first week post-injection, whereas most human RPOA (9, 10) and 13/19 cases in our study manifested at least 6 months after treatment initiation.

A limitation of our descriptive analysis is the inherent subjectivity associated with expert judgment. To mitigate potential bias, the adjudication panel primarily comprised veterinarians with a shared interest in advancing pain management for companion animals. Importantly, none had financial ties to veterinary pharmaceutical companies. Having mitigated bias, and recognizing the inherent subjectivity in data analysis and interpretation, we prioritized *data presentation* to facilitate independent judgment by readers, regardless of their level of expertise. Another acknowledged limitation of our study pertains to the FDA’s guidance that AE signal detection should primarily serve as a *hypothesis-generating tool*. Accordingly, our exploratory study was designed to identify potential safety signals rather than provide a comprehensive safety profile. As such, it cannot address specific questions like the impact of NSAID co-administration on MSAER risk. However, we believe these findings offer valuable insights and will stimulate further investigation.

Our study highlights an important weakness in the current pharmacovigilance system: the lack of comprehensive terminology for accurately capturing serious AEs. The absence of RPOA as a diagnostic term in VeDDRA is of particular concern, potentially leading to a substantial underestimation of MSAERs. Without a specific term, these events may be misclassified as manifestations of the underlying condition being treated (e.g., “arthritis” or “lameness”), obscuring the true incidence and severity of ADRs. To address this deficiency and enhance data quality, we formally requested the addition of the term “RPOA” to VeDDRA (16). Our proposed definition, “joint pathology falling well outside the natural history of OA,” leverages the expertise and clinical judgment of reporting veterinarians with direct access to patient data.

An FDA panelist involved in the adjudication of humanized aNGFmAbs eloquently summarised our current belief: “All parties agree that the use of aNGFmabs is effective, but they are associated with a unique, rapidly progressing form of OA…and we can only speculate as to its causes (8).” In animals, just as in humans, the goal of effective pain management is paramount. However, we must also ensure that our therapeutic interventions do not inadvertently exacerbate the underlying condition. To uphold the highest standard of care for companion animals, we hope to apply the same rigorous scrutiny to veterinary mAbs as was employed in human healthcare.

## Data Availability

The original contributions presented in the study are included in the article/Supplementary material.

## Supplementary material

SUPPLEMENTARY VIDEO 1 Demonstration of the EudraVigilance database MSAER search strategy. The database can be accessed at: https://www.adrreports.eu/vet/en/search.html# (Accessed September 18–23, 2024 and January 1–10, 2025).

SUPPLEMENTARY VIDEO 2 Narrated slideshow used by the independent adjudication panel.

SUPPLEMENTARY TABLE S1 789 musculoskeletal adverse event reports to Librela (May 2021–December 2024).

SUPPLEMENTARY FIGURE S1 Case 1—AER translation error. The attending specialist filed an AER specifying their suspicion of RPOA. The MAH filed a report with an incorrect diagnosis of septic arthritis.

SUPPLEMENTARY FIGURE S2 Case 2—AER translation error. The MAH filed a report specifying “overdose” but the administered dose (0.6 mg/kg) was in the recommended range (38).

SUPPLEMENTARY FIGURE S3 Case 4—AER translation error. A 9.3-year-old Labrador Retriever sustained a pathological elbow fracture. The attending specialist filed an AER to the VMD specifying their suspicion of RPOA. This report was shared with the MAH, who filed a report for non-serious “arthritis”, with a listed outcome of recovered/resolving.

SUPPLEMENTARY FIGURE S4 Case 10—AER translation error. A 10-year-old GSD required hindlimb amputation to manage erosive tarsometatarsal OA. The attending specialist filed an AER to the VMD including the result of the histopathological analysis (which was consistent with RPOA). The MAH filed a report stating that the AE was recovered/resolving following “digit amputation”.

SUPPLEMENTARY FIGURE S5 Case 11—AER translation error. The attending specialist filed an AER to the VMD specifying a diagnosis of ongoing RPOA. The MAH filed a report for non-serious arthritis which was recovered/resolving.

SUPPLEMENTARY FIGURE S6 Case 12—AER translation error. The attending specialist filed an AER to the VMD specifying a diagnosis of severe RPOA. The MAH filed a report for a non-severe bone and joint disorder which was recovered/resolving. The MAH filed a report specifying “overdose” but the administered dose (0.7 mg/kg) was in the recommended range (38).

SUPPLEMENTARY FIGURE S7 Case 13—AER translation error. The attending specialist filed an AER specifying their suspicion of RPOA. The MAH filed a report with an erroneous diagnosis of osteosarcoma.

SUPPLEMENTARY FIGURE S8 Case 15—AER translation error. A 9.5-year-old English Bull Terrier developed erosive arthritis in previously normal hock joints. The MAH filed a report with a diagnosis of non-serious swollen joint which was recovered/resolving.

SUPPLEMENTARY FIGURE S9 Case 18—AER translation error. A 6-year-old Australian Shepherd developed bilateral stifle joint luxations and fibular fractures following 8 doses of Librela. The MAH filed an AER designating this reaction as not serious.

## Glossary

ADR: Adverse drug reaction
AE: adverse event
AER: adverse event report
aNGFmAb: anti-NGF monoclonal antibody
CT: computed tomography
EVD: EudraVigilance database
EEBVS: European Board of Veterinary Specialisation
EMA: European Medicines Agency
FDA: Food and Drug Administration
HCF: humeral condylar fracture
HIF: humeral intracondylar fissure
MAH: marketing authorization holder
MRI: magnetic resonance imaging
MCP: medial coronoid process
MSAE: musculoskeletal adverse event
MSAER: musculoskeletal adverse event report
NGF: nerve growth factor
NSAIDs: non-steroidal anti-inflammatory drugs
OA: osteoarthritis
RPOA: rapidly progressive osteoarthritis
REMS: risk evaluation and mitigation strategy
TPLO: tibial plateau leveling osteotomy
VeDDRA: Veterinary Dictionary for Drug-Related Adverse Reactions
VMD: Veterinary Medicines Directorate
