# Supplementary material for: Musculoskeletal adverse events in dogs receiving bedinvetmab (Librela)
Source: Front Vet Sci. 2025 May 9;12:1581490. doi: 10.3389/fvets.2025.1581490 (PMC12100767; doi:10.3389/fvets.2025.1581490)
Supplement: SUPPLEMENTARY TABLE S1 — 789 musculoskeletal adverse event reports to Librela (May 2021–December 2024). [file Table_1.pdf]

Date	Reference	Reaction	Sender	Serious	Outcome	Other medications
20 May 2021	GBR-ZOETISPV-2021-UK-00926	Tendon rupture	Zoetis (Belgium)	Yes	Unknown	No
7 Jun 2021	GBR-ZOETISPV-2021-UK-01051	Fracture; joint cartilage disorder (NOS)	Zoetis (Belgium)	Yes	Unknown	No
20 Jun 2021	ESP-ZOETISPV-2024-ES-00542	Lameness; lack of efficacy	Zoetis (Belgium)	No	Recovered/resolving	No
10 Aug 2021	DEU-ZOETISPV-2021-DE-00236	Fracture	Paul Ehrlich Institute	Yes	Unknown	No
19 Aug 2021	DEU-ZOETISPV-2021-DE-00790	Non-weight bearing lameness	Zoetis (Belgium)	No	Unknown	No
19 Aug 2021	GBR-ZOETISPV-2021-UK-01718	Ligament rupture	Zoetis (Belgium)	Yes	Unknown	No
1 Sep 2021	DEU-ZOETISPV-2021-DE-00720	Fracture	Paul Ehrlich Institute	Yes	Unknown	No
7 Sep 2021	DEU-ZOETISPV-2021-DE-00937	Musculoskeletal pain; unable to rise; difficulty walking	Zoetis (Belgium)	Yes	Recovered/resolving	No
13 Sep 2021	GBR-BIAHPV1P-21UK001423	Arthritis	Boehringer Ingelheim	No	Recovered/resolving	Meloxicam
20 Sep 2021	DE-BVL-2021-16797	Swollen joint	Paul Ehrlich Institute	No	Recovered/resolving	No
23 Sep 2021	FRA-ZOETISPV-2021-FR-00616	Ligament rupture	NVMA France	Yes	Ongoing	No
14 Oct 2021	GBR-ZOETISPV-2021-UK-02210	Tendon injury, swollen joint, tendon injury	Zoetis (Belgium)	Yes	Unknown	No
2 Nov 2021	GBR-ZOETISPV-2021-UK-02367	Fracture; lameness	Zoetis (Belgium)	Yes	Euthanasia	No
10 Nov 2021	GBR-ZOETISPV-2021-UK-02471	Musculoskeletal neoplasia; joint swelling	Zoetis (Belgium)	Yes	Unknown	No
13 Jan 2022	DEU-ZOETISPV-2021-DE-00582	Ligament rupture	Paul Ehrlich Institute	Yes	Unknown	No
22 Jan 2022	NLD-ZOETISPV-2022-NL-00003	Joint ligament disorder	Dutch VMA	Yes	Euthanasia	No
2 Feb 2022	GBR-ZOETISPV-2022-UK-00194	Musculoskeletal neoplasia; joint swelling	Zoetis (Belgium)	Yes	Unknown	No
7 Feb 2022	GRC-ZOETISPV-2022-GR-00002	Joint oedema; swollen feet	Zoetis (Belgium)	No	Unknown	No
10 Feb 2022	ESP-ZOETISPV-2021-ES-00205	Joint inflammation	Zoetis (Belgium)	No	Recovered/resolving	No
10 Feb 2022	ESP-ZOETISPV-2021-ES-00508	Swollen joint	Zoetis (Belgium)	No	Unknown	No
10 Feb 2022	FRA-ZOETISPV-2021-FR-00358	Joint effusion; localised oedema	Zoetis (Belgium)	No	Ongoing	No
10 Feb 2022	GBR-ZOETISPV-2021-UK-00785	Joint swelling, lameness	Zoetis (Belgium)	No	Recovered/resolving	Meloxicam
10 Feb 2022	GBR-ZOETISPV-2021-UK-01211	Swollen joint	Zoetis (Belgium)	No	Unknown	No
10 Feb 2022	UK-VMD-04181/21	Joint effusion	Zoetis (Belgium)	No	Recovered/resolving	No
10 Feb 2022	DEU-ZOETISPV-2021-DE-00518	Lameness	Zoetis (Belgium)	No	Unknown	No
10 Feb 2022	GBR-ZOETISPV-2021-UK-00744	Lameness	Zoetis (Belgium)	No	Unknown	No
10 Feb 2022	GBR-ZOETISPV-2021-UK-00781	Lameness	Zoetis (Belgium)	No	Unknown	No
10 Feb 2022	GBR-ZOETISPV-2021-UK-00930	Lameness	Zoetis (Belgium)	No	Unknown	No
10 Feb 2022	BEL-ZOETISPV-2021-BE-00053	Ligament rupture	Zoetis (Belgium)	No	Recovered/resolving	No
10 Feb 2022	GBR-ZOETISPV-2021-UK-00743	Septic arthritis	Zoetis (Belgium)	No	Unknown	No

Date	Reference	Reaction	Sender	Serious	Outcome	Other medications
10 Feb 2022	GBR-ZOETISPV-2021-UK-01346	Difficulty rising	Zoetis (Belgium)	No	Recovered/resolving	No
10 Feb 2022	GBR-ZOETISPV-2021-UK-01469	Difficulty rising	Zoetis (Belgium)	No	Unknown	Carprofen
11 Feb 2022	GBR-ZOETISPV-2021-UK-01812	Bone and joint disorder	Zoetis (Belgium)	No	Unknown	No
11 Feb 2022	GBR-ZOETISPV-2021-UK-02875	Fracture	Zoetis (Belgium)	No	Unknown	No
11 Feb 2022	ESP-ZOETISPV-2021-ES-00862	Joint pain; bone and joint disorder	Zoetis (Belgium)	No	Recovered/resolving	No
11 Feb 2022	ESP-ZOETISPV-2021-ES-00828	Limping	Zoetis (Belgium)	No	Recovered/resolving	No
11 Feb 2022	GBR-ZOETISPV-2021-UK-01767	Lameness; bone and joint disorder	Zoetis (Belgium)	No	Unknown	No
11 Feb 2022	GBR-ZOETISPV-2021-UK-01975	Gait abnormality	Zoetis (Belgium)	No	Unknown	No
11 Feb 2022	GBR-ZOETISPV-2021-UK-02511	Lameness	Zoetis (Belgium)	No	Unknown	No
11 Feb 2022	GBR-ZOETISPV-2022-UK-00039	Limping	Zoetis (Belgium)	No	Unknown	Meloxicam
11 Feb 2022	BEL-ZOETISPV-2021-BE-00067	Ligament rupture	Zoetis (Belgium)	No	Recovered/resolving	No
11 Feb 2022	GBR-ZOETISPV-2021-UK-02326	Joint ligament disorder	Zoetis (Belgium)	No	Unknown	No
11 Feb 2022	GBR-ZOETISPV-2021-UK-02328	Ligament rupture	Zoetis (Belgium)	No	Recovered/resolving	Meloxicam
11 Feb 2022	FRA-ZOETISPV-2021-FR-00721	Polyarthritis; hyperthermia	Zoetis (Belgium)	No	Unknown	No
11 Feb 2022	GBR-ZOETISPV-2021-UK-02219	Stiffness and pain	Zoetis (Belgium)	No	Unknown	No
11 Feb 2022	DEU-ZOETISPV-2021-DE-01008	Joint swelling, lameness	Zoetis (Belgium)	No	Unknown	No
11 Feb 2022	ESP-ZOETISPV-2021-ES-00690	Lameness	Zoetis (Belgium)	No	Unknown	No
11 Feb 2022	GBR-ZOETISPV-2021-UK-01159	Joint swelling, lameness	Zoetis (Belgium)	No	Recovered/resolving	No
11 Feb 2022	GBR-ZOETISPV-2021-UK-01824	Joint swelling, joint pain, lameness	Zoetis (Belgium)	No	Unknown	Firocoxib
11 Feb 2022	GBR-ZOETISPV-2021-UK-01942	Joint swelling	Zoetis (Belgium)	No	Unknown	Pentosan polysulphate
11 Feb 2022	GBR-ZOETISPV-2021-UK-02393	Joint swelling	Zoetis (Belgium)	No	Unknown	No
11 Feb 2022	NLD-ZOETISPV-2021-NL-00398	Swollen joint	Zoetis (Belgium)	No	Resolved with sequelae	No
12 Feb 2022	ITA-ZOETISPV-2021-IT-00116	Musculoskeletal disorder; pain; lack of efficacy	Zoetis (Belgium)	No	Recovered/resolving	No
24 Feb 2022	DEU-ZOETISPV-2021-DE-00752	Bone and joint disorder; lack of efficacy	German FDA	No	Recovered/resolving	Cimicoxib
25 Feb 2022	FRA-ZOETISPV-2022-FR-00065	Lameness	French VMA	No	Recovered/resolving	No
7 Mar 2022	FRA-ZOETISPV-2022-FR-00123	Joint swelling	Zoetis (Belgium)	No	Resolved with sequelae	No
9 Mar 2022	GBR-ZOETISPV-2022-XI-00007	Joint swelling, abnormal radiograph finding	Zoetis (Belgium)	No	Unknown	No
10 Mar 2022	IRL-ZOETISPV-2022-IE-00027	Lameness; muscle atrophy	Zoetis (Belgium)	Yes	Resolved with sequelae	No
10 Mar 2022	FRA-FRAANMVF-202200847	Musculoskeletal neoplasia; joint inflammation	French VMA	Yes	Unknown	Cimicoxib
14 Mar 2022	BEL-ZOETISPV-2022-BE-00036	Polyarthritis; swollen limb	Zoetis (Belgium)	Yes	Recovered/resolving	No

Date	Reference	Reaction	Sender	Serious	Outcome	Other medications
17 Mar 2022	DEU-ZOETISPV-2022-DE-00221	Joint swelling	Zoetis (Belgium)	Yes	Unknown	No
18 Mar 2022	GBR-ZOETISPV-2022-UK-00477	Joint stiffness; malaise	Zoetis (Belgium)	No	Recovered/resolving	No
18 Mar 2022	DEU-ZOETISPV-2021-DE-01272	Difficulty rising and walking	Zoetis (Belgium)	No	Unknown	No
31 Mar 2022	DEU-ZOETISPV-2022-DE-00267	Joint swelling, arthropathy	Zoetis (Belgium)	Yes	Fatal	No
7 Apr 2022	DEU-ZOETISPV-2022-DE-00326	Joint pain; reluctant to move; partial lack of efficacy	Zoetis (Belgium)	Yes	Unknown	No
13 Apr 2022	FIN-ZOETISPV-2022-FI-00029	Joint inflammation, joint pain	Zoetis (Belgium)	No	Unknown	No
14 Apr 2022	GBR-ZOETISPV-2021-UK-02924	Tendon rupture; implant infection	Zoetis (Belgium)	Yes	Euthanasia	No
21 Apr 2022	DEU-ZOETISPV-2022-DE-00285	Ligament rupture; lack of efficacy	German VMA	No	Recovered/resolving	Meloxicam
5 May 2022	FRA-ZOETISPV-2022-FR-00285	Lameness; hypersalivation	Zoetis (Belgium)	No	Recovered/resolving	No
11 May 2022	ESP-ZOETISPV-2022-ES-00257	Polyarthritis	Zoetis (Belgium)	No	Unknown	No
11 May 2022	ESP-ZOETISPV-2022-ES-00258	Joint inflammation	Zoetis (Belgium)	No	Unknown	No
18 May 2022	DEU-ZOETISPV-2022-DE-00473	Polyarthritis	Zoetis (Belgium)	No	Recovered/resolving	No
8 Jun 2022	GBR-ZOETISPV-2022-UK-01361	Joint oedema	Zoetis (Belgium)	No	Unknown	No
17 Jun 2022	GBR-ZOETISPV-2022-XI-00036	Swollen joint, limb non-weight-bearing	Zoetis (Belgium)	No	Unknown	No
20 Jun 2022	SWE-ZOETISPV-2022-SE-00068	Swollen joint	Zoetis (Belgium)	No	Unknown	No
23 Jun 2022	GBR-ZOETISPV-2022-UK-01374	Non-weight bearing lameness; reluctant to move	Zoetis (Belgium)	No	Unknown	No
24 Jun 2022	DEU-ZOETISPV-2022-DE-00632	Joint swelling	Zoetis (Belgium)	No	Unknown	No
29 Jun 2022	GBR-ZOETISPV-2022-UK-01501	Joint swelling, abnormal radiograph finding	Zoetis (Belgium)	No	Unknown	No
11 Jul 2022	DEU-ZOETISPV-2022-DE-00713	Joint swelling	Zoetis (Belgium)	Yes	Fatal	No
29 Jul 2022	GBR-ZOETISPV-2022-UK-00788	Joint effusion, abnormal radiograph finding	Zoetis (Belgium)	No	Unknown	Meloxicam
10 Aug 2022	GBR-ZOETISPV-2022-UK-01721	Lameness and pain	Zoetis (Belgium)	No	Unknown	No
17 Aug 2022	FRA-FRAANMVF-202202764	Musculoskeletal pain	French VMA	No	Recovered/resolving	No
14 Sep 2022	SWE-ZOETISPV-2022-SE-00108	Arthritis; lack of efficacy	VMD (Zoetis Belgium)	Yes	Unknown	No
26 Sep 2022	FIN-ZOETISPV-2022-FI-00095	Bone and joint disorder; polydipsia	Zoetis (Belgium)	No	Recovered/resolving	No
26 Sep 2022	DEU-ZOETISPV-2022-DE-01012	Hyperextension	Zoetis (Belgium)	No	Unknown	No
3 Oct 2022	ITA-ZOETISPV-2022-IT-00151	Lameness; depression; vomiting	Zoetis (Belgium)	No	Recovered/resolving	No
6 Oct 2022	GBR-ZOETISPV-2022-UK-02441	Joint pain, unable to rise	Zoetis (Belgium)	No	Unknown	No
10 Oct 2022	IRL-ZOETISPV-2022-IE-00161	Stiffness; limb tremor	Zoetis (Belgium)	No	Recovered/resolving	No
17 Oct 2022	DEU-ZOETISPV-2022-DE-01119	Gait abnormality	Zoetis (Belgium)	No	Unknown	No
18 Oct 2022	GBR-ZOETISPV-2022-UK-02599	Bone and joint disorder	Zoetis (Belgium)	No	Unknown	No

Date	Reference	Reaction	Sender	Serious	Outcome	Other medications
18 Oct 2022	GBR-ZOETISPV-2022-UK-02599	Bone and joint disorder	Zoetis (Belgium)	No	Unknown	No
19 Oct 2022	DEU-ZOETISPV-2022-DE-01135	Gait abnormality	Zoetis (Belgium)	No	Unknown	No
26 Oct 2022	FRA-ZOETISPV-2022-FR-00711	Joint oedema and pain	Zoetis (Belgium)	No	Unknown	No
31 Oct 2022	GBR-ZOETISPV-2022-UK-02911	Bone and joint disorder; abnormal test result	Zoetis (Belgium)	No	Unknown	No
31 Oct 2022	GBR-ZOETISPV-2022-UK-02906	Bone and joint disorder; abnormal test result	Zoetis (Belgium)	No	Recovered/resolving	No
11 Nov 2022	GBR-ZOETISPV-2022-UK-03077	Joint ligament rupture	Zoetis (Belgium)	No	Unknown	No
14 Nov 2022	GBR-VMDDEFRA-02896/22	Difficulty rising and standing; gait abnormality	VMD (Zoetis Belgium)	No	Recovered/resolving	No
22 Nov 2022	GBR-ZOETISPV-2022-UK-03026	Immune-mediated polyarthritis	Zoetis (Belgium)	Yes	Unknown	No
23 Nov 2022	DEU-ZOETISPV-2022-DE-01208	Lameness	Zoetis (Belgium)	No	Unknown	No
25 Nov 2022	GBR-ZOETISPV-2022-UK-03068	Joint swelling, pyrexia	Zoetis (Belgium)	No	Unknown	No
25 Nov 2022	GBR-ZOETISPV-2022-UK-03082	Bone and joint disorder	Zoetis (Belgium)	No	Unknown	No
29 Nov 2022	GBR-ZOETISPV-2022-UK-01597	Joint swelling, arthritis	Zoetis (Belgium)	No	Unknown	No
29 Dec 2022	DEU-ZOETISPV-2022-DE-01386	Tendon rupture	Zoetis (Belgium)	Yes	Recovered/resolving	No
2 Jan 2023	GBR-VMDDEFRA-03242/22	Limping	VMD (Zoetis Belgium)	No	Recovered/resolving	No
3 Jan 2023	IRL-ZOETISPV-2022-IE-00222	Joint pain, joint stiffness, reluctant to move	Zoetis (Belgium)	No	Recovered/resolving	No
10 Jan 2023	GBR-ZOETISPV-2022-UK-03088	Joint swelling	Zoetis (Belgium)	No	Recovered/resolving	Meloxicam
16 Jan 2023	DNK-DNKMEDAG-V20230002	Joint pain; bone and joint disorder	Danish Medicines Agency	No	Unknown	No
19 Jan 2023	GBR-ZOETISPV-2023-UK-00021	Limb oedema; non-weight bearing lameness	Zoetis (Belgium)	No	Unknown	Prednisolone
19 Jan 2023	SWE-ZOETISPV-2023-SE-00003	Lameness; difficulty getting into a car	Zoetis (Belgium)	No	Unknown	No
14 Feb 2023	DEU-ZOETISPV-2023-DE-00087	Swollen joint	Zoetis (Belgium)	Yes	Resolved with sequelae	No
16 Feb 2023	GBR-ZOETISPV-2023-UK-00235	Limb swelling; lameness	Zoetis (Belgium)	No	Unknown	No
21 Feb 2023	GBR-ZOETISPV-2023-UK-00282	Musculoskeletal neoplasia; joint swelling	Zoetis (Belgium)	Yes	Unknown	Lokivetmab
23 Feb 2023	DEU-ZOETISPV-2023-DE-00120	Arthritis; partial lack of efficiency	Zoetis (Belgium)	Yes	Recovered/resolving	Firocoxib
2 Mar 2023	DEU-ZOETISPV-2023-DE-00154	Bursitis	Zoetis (Belgium)	No	Resolved with sequelae	No
3 Mar 2023	DEU-ZOETISPV-2023-DE-00167	Ligament rupture	Zoetis (Belgium)	Yes	Recovered/resolving	No
3 Mar 2023	DEU-ZOETISPV-2023-DE-00166	Arthrosis	Zoetis (Belgium)	Yes	Euthanasia	No
6 Mar 2023	SWE-SWEMEDAG-2023-00076	Lameness	Swedish VMA	No	Recovered/resolving	No
6 Mar 2023	DNK-ZOETISPV-2023-DK-00009	Polyarthritis	Zoetis (Belgium)	No	Recovered/resolving	Cyclosporine
8 Mar 2023	GBR-ZOETISPV-2023-XI-00010	Joint ligament disorder; joint swelling	Zoetis (Belgium)	No	Unknown	Cimicoxib
9 Mar 2023	CHE-ZOETISPV-2023-CH-00007	Ligament rupture	Zoetis (Belgium)	Yes	Recovered/resolving	No

Date	Reference	Reaction	Sender	Serious	Outcome	Other medications
13 Mar 2023	POL-ZOETISPV-2023-PL-00020	Cartilage degeneration; abnormal test result	Zoetis (Belgium)	No	Recovered/resolving	No
13 Mar 2023	DEU-ZOETISPV-2023-DE-00157	Gait abnormality; partial lack of efficacy	Zoetis (Belgium)	No	Unknown	No
13 Mar 2023	AUS-ZOETISPV-2023-AU-01923	Torn ligament; hyperexcitation	Zoetis (Belgium)	No	Unknown	No
13 Mar 2023	AUS-ZOETISPV-2023-AU-01926	Torn ligament; hyperexcitation	Zoetis (Belgium)	No	Unknown	No
16 Mar 2023	GBR-ZOETISPV-2023-UK-00265	Joint swelling, bone and joint disorder	Zoetis (Belgium)	No	Unknown	Meloxicam
20 Mar 2023	GBR-ZOETISPV-2023-UK-00707	Joint ligament disorder; joint swelling	Zoetis (Belgium)	No	Unknown	Meloxicam
23 Mar 2023	POL-ZOETISPV-2023-PL-00026	Musculoskeletal neoplasia	Zoetis (Belgium)	Yes	Unknown	No
7 Apr 2023	FRA-ZZELANCO-FR2023_000131	Arthritis; dental calculus; tiredness	Elanco	No	Unknown	No
14 Apr 2023	GBR-ZOETISPV-2023-UK-01085	Joint swelling	Zoetis (Belgium)	No	Unknown	Tramadol
18 Apr 2023	GBR-VMDDEFRA-00040/23	Fracture; joint swelling	VMD (Zoetis Belgium)	Yes	Recovered/resolving	No
25 Apr 2023	GBR-ZOETISPV-2023-UK-01102	Musculoskeletal neoplasia; fracture	Zoetis (Belgium)	Yes	Unknown	No
15 May 2023	POL-ZOETISPV-2023-PL-00061	Hyperextension	Zoetis (Belgium)	No	Recovered/resolving	No
17 May 2023	CAN-ZOETISPV-2023-CA-01372	Torn ligament	Zoetis (Belgium)	No	Recovered/resolving	No
25 May 2023	ESP-ZOETISPV-2023-ES-00322	Bone and joint disorder; joint stiffness	Zoetis (Belgium)	Yes	Recovered/resolving	No
30 May 2023	DEU-ZOETISPV-2023-DE-00528	Joint swelling, joint pain	Zoetis (Belgium)	No	Recovered/resolving	No
5 Jun 2023	AUS-ZOETISPV-2023-AU-01653	Lameness; partial lack of efficacy	Zoetis (Belgium)	No	Unknown	No
6 Jun 2023	DEU-ZOETISPV-2023-DE-00569	Difficulty going up and down stairs; lameness	Zoetis (Belgium)	No	Recovered/resolving	No
6 Jun 2023	CAN-ZOETISPV-2023-CA-01701	Musculoskeletal pain	Zoetis (Belgium)	No	Resolved with sequelae	No
7 Jun 2023	GBR-ZOETISPV-2023-UK-01753	Joint swelling	Zoetis (Belgium)	No	Recovered/resolving	No
12 Jun 2023	DEU-ZOETISPV-2023-DE-00581	Joint swelling, arthritis	Zoetis (Belgium)	No	Unknown	No
13 Jun 2023	ESP-ZOETISPV-2023-ES-00383	Bone and joint disorder, partial lack of efficacy	Zoetis (Belgium)	No	Unknown	No
13 Jun 2023	FIN-ZOETISPV-2023-FI-00083	Abnormal radiographic finding; cartilage degeneration	Zoetis (Belgium)	Yes	Fatal	Carprofen
15 Jun 2023	ISR-ZOETISPV-2023-IL-00035	Musculoskeletal neoplasia; joint oedema	Zoetis (Belgium)	Yes	Unknown	Carprofen
20 Jun 2023	CAN-ZOETISPV-2023-CA-01977	Difficulty going up and down stairs	Zoetis (Belgium)	No	Unknown	No
27 Jun 2023	NLD-ZOETISPV-2023-NL-00238	Joint swelling, lameness	Zoetis (Belgium)	No	Recovered/resolving	Grapiprant
28 Jun 2023	FIN-ZOETISPV-2023-FI-00104	Limping; partial lack of efficacy	Zoetis (Belgium)	No	Recovered/resolving	No
30 Jun 2023	GBR-DECHRALT-2023-07156	Arthritis; heart murmur; weight loss	Dechra	No	Unknown	Thyroxine
3 Jul 2023	CAN-ZOETISPV-2023-CA-02195	Joint pain; lameness	Zoetis (Belgium)	No	Unknown	No
3 Jul 2023	NLD-ZOETISPV-2023-NL-00249	Joint pain	Zoetis (Belgium)	No	Recovered/resolving	No
11 Jul 2023	FRA-FRAANMVF-202303058	Arthrosis (joint cartilage disorder)	French VMA	Yes	Unknown	No

Date	Reference	Reaction	Sender	Serious	Outcome	Other medications
17 Jul 2023	CAN-ZOETISPV-2023-CA-02496	Non-weight bearing lameness	Zoetis (Belgium)	No	Unknown	Grapiprant
17 Jul 2023	AUS-ZOETISPV-2023-AU-00821	Ligament rupture	Zoetis (Belgium)	No	Unknown	No
17 Jul 2023	GBR-ZOETISPV-2023-UK-02299	Stiffness	Zoetis (Belgium)	No	Recovered/resolving	Grapiprant
27 Jul 2023	FRA-FRAANMVF-202303686	Joint oedema	NVMA France	No	Ongoing	No
28 Jul 2023	FIN-FINAMVET-20230152	Limping; lack of efficacy	Finnish Medicines Agency	No	Recovered/resolving	No
28 Jul 2023	FIN-FINAMVET-20230151	Stiffness; lack of efficacy	Zoetis (Belgium)	No	Recovered/resolving	No
31 Jul 2023	ESP-ZOETISPV-2023-ES-00585	Arthritis	Zoetis (Belgium)	No	Recovered/resolving	Firocoxib
31 Jul 2023	AUS-ZOETISPV-2023-AU-00918	Torn ligament	Zoetis (Belgium)	Yes	Unknown	No
31 Jul 2023	GBR-MERCKMSD-2023-UK-03326	Immune-mediated polyarthritis	Intervet	Yes	Recovered/resolving	Osaterane
3 Aug 2023	NLD-ZOETISPV-2023-NL-00305	Joint swelling	Zoetis (Belgium)	Yes	Recovered/resolving	Carprofen
3 Aug 2023	NLD-ZOETISPV-2023-NL-00307	Joint effusion, bone and joint disorder	Zoetis (Belgium)	No	Recovered/resolving	Carprofen
7 Aug 2023	ESP-ZOETISPV-2023-ES-00599	Immune-mediated polyarthritis	Zoetis (Belgium)	Yes	Recovered/resolving	Mavacoxib
8 Aug 2023	AUS-ZOETISPV-2023-AU-01041	Fracture	Zoetis (Belgium)	No	Recovered/resolving	Firocoxib
9 Aug 2023	DEU-ZOETISPV-2023-DE-00843	Difficulty rising; lack of efficacy	Zoetis (Belgium)	No	Unknown	No
9 Aug 2023	AUS-ZOETISPV-2023-AU-00993	Ligament rupture	Zoetis (Belgium)	No	Recovered/resolving	No
9 Aug 2023	AUS-ZOETISPV-2023-AU-00994	Ligament rupture	Zoetis (Belgium)	No	Unknown	No
10 Aug 2023	AUS-ZOETISPV-2023-AU-01001	Fracture	Zoetis (Belgium)	Yes	Recovered/resolving	No
15 Aug 2023	FRA-ZOETISPV-2023-FR-00344	Polyarthritis	Zoetis (Belgium)	Yes	Euthanasia	No
15 Aug 2023	FRA-ZOETISPV-2023-FR-00345	Polyarthritis	Zoetis (Belgium)	Yes	Euthanasia	No
15 Aug 2023	FRA-ZOETISPV-2023-FR-00346	Polyarthritis	Zoetis (Belgium)	Yes	Euthanasia	Meloxicam
15 Aug 2023	FRA-ZOETISPV-2023-FR-00347	Polyarthritis	Zoetis (Belgium)	Yes	Euthanasia	Benazapril
21 Aug 2023	JPN-ZOETISPV-2023-JP-01060	Difficulty rising	Zoetis (Belgium)	No	Recovered/resolving	No
28 Aug 2023	FRA-FRAANMVF-202304509	Limb swelling; arthrosis	French VMA	Yes	Euthanasia	Prednisolone
30 Aug 2023	GBR-ZOETISPV-2023-UK-02924	Limping	Zoetis (Belgium)	No	Recovered/resolving	Meloxicam
4 Sep 2023	CAN-ZOETISPV-2023-CA-03297	Lameness; trembling	Zoetis (Belgium)	No	Recovered/resolving	No
6 Sep 2023	CAN-ZOETISPV-2023-CA-03359	Ligament rupture; abnormal radiograph finding	Zoetis (Belgium)	No	Recovered/resolving	Pentosan polysulphate
6 Sep 2023	CAN-ZOETISPV-2023-CA-03362	Limb collapse	Zoetis (Belgium)	Yes	Unknown	No
7 Sep 2023	GBR-ZOETISPV-2023-UK-03227	Non-weight bearing lameness	Zoetis (Belgium)	No	Recovered/resolving	No
8 Sep 2023	CAN-ZOETISPV-2023-CA-03143	Joint pain, gait abnormality	Zoetis (Belgium)	Yes	Fatal	No
11 Sep 2023	GBR-ZOETISPV-2023-UK-03120	Joint swelling, bone and joint disorder, arthritis	Zoetis (Belgium)	No	Unknown	No

Date	Reference	Reaction	Sender	Serious	Outcome	Other medications
12 Sep 2023	FRA-FRAANMVF-202304598	Lameness	French VMA	No	Recovered/resolving	No
14 Sep 2023	ESP-ZOETISPV-2023-ES-00731	Ligament rupture	Zoetis (Belgium)	Yes	Fatal	No
14 Sep 2023	ESP-ZOETISPV-2023-ES-00732	Ligament rupture	Zoetis (Belgium)	No	Recovered/resolving	No
14 Sep 2023	ESP-ZOETISPV-2023-ES-00733	Ligament rupture; partial lack of efficacy	Zoetis (Belgium)	No	Recovered/resolving	No
14 Sep 2023	ESP-ZOETISPV-2023-ES-00734	Ligament rupture	Zoetis (Belgium)	No	Recovered/resolving	No
14 Sep 2023	CAN-ZOETISPV-2023-CA-03538	Immune-mediated polyarthritis	Zoetis (Belgium)	Yes	Recovered/resolving	No
15 Sep 2023	GBR-ZOETISPV-2023-XI-00023	Joint effusion, bone and joint disorder	Zoetis (Belgium)	No	Unknown	Robenacoxib
18 Sep 2023	AUS-ZOETISPV-2023-AU-01360	Ligament rupture; abnormal radiograph finding	Zoetis (Belgium)	Yes	Recovered/resolving	No
21 Sep 2023	FIN-ZOETISPV-2023-FI-00166	Lameness; partial lack of efficacy	Zoetis (Belgium)	Yes	Euthanasia	Meloxicam
21 Sep 2023	AUS-ZOETISPV-2023-AU-01153	Rhabdomyosarcoma; abnormal radiograph finding	Zoetis (Belgium)	Yes	Euthanasia	No
25 Sep 2023	ISR-ZOETISPV-2023-IL-00096	Lameness; trembling; partial lack of efficacy	Zoetis (Belgium)	No	Recovered/resolving	No
25 Sep 2023	CAN-ZOETISPV-2023-CA-03892	Difficulty going up and down stairs; sleepiness	Zoetis (Belgium)	No	Unknown	No
27 Sep 2023	GBR-ZOETISPV-2023-UK-03537	Reluctant to move; stiffness; walking difficulty	Zoetis (Belgium)	No	Unknown	No
2 Oct 2023	AUT-ZOETISPV-2023-AT-00063	Polyarthritis; joint ligament disorder	Zoetis (Belgium)	No	Resolved with sequelae	No
2 Oct 2023	GBR-ZOETISPV-2023-UK-03348	Joint swelling	Zoetis (Belgium)	No	Recovered/resolving	No
3 Oct 2023	NLD-ZOETISPV-2023-NL-00247	Myofibrosarcoma; septic arthritis	Zoetis (Belgium)	Yes	Unknown	Cyclosporine
10 Oct 2023	AUS-ZOETISPV-2023-AU-01555	Hyperextension	Zoetis (Belgium)	Yes	Unknown	Pentosan polysulphate
11 Oct 2023	USA-ZOETISPV-2023-US-34803	Ligament rupture	Zoetis (Belgium)	No	Recovered/resolving	No
11 Oct 2023	CAN-ZOETISPV-2023-CA-03947	Osteosarcoma; joint swelling	Zoetis (Belgium)	Yes	Resolved with sequelae	No
16 Oct 2023	GBR-ZOETISPV-2023-UK-03598	Joint pain; lameness; decreased activity	Zoetis (Belgium)	No	Unknown	No
16 Oct 2023	SWE-SWEMEDAG-2023-00393	Joint pain; lameness; reluctant to move	Zoetis (Belgium)	No	Recovered/resolving	No
17 Oct 2023	BEL-ZOETISPV-2023-BE-00185	Swollen joint, joint stiffness	Zoetis (Belgium)	No	Recovered/resolving	Oclacitinib
18 Oct 2023	AUS-ZOETISPV-2023-AU-01314	Gait abnormality	Zoetis (Belgium)	No	Unknown	No
19 Oct 2023	GBR-ZOETISPV-2023-UK-03388	Fracture; joint swelling; arthritis	Zoetis (Belgium)	Yes	Euthanasia	No
19 Oct 2023	GBR-ZOETISPV-2023-UK-03388	Fracture; joint swelling; arthritis	Zoetis (Belgium)	Yes	Euthanasia	No
24 Oct 2023	CAN-ZOETISPV-2023-CA-04154	Joint swelling	Zoetis (Belgium)	No	Recovered/resolving	No
24 Oct 2023	GBR-ZOETISPV-2023-UK-03679	Abnormal radiographic finding; bone and joint disorder	Zoetis (Belgium)	No	Unknown	Meloxicam, tramadol
24 Oct 2023	USA-ZOETISPV-2023-US-37259	Ligament rupture; unable to rise	Zoetis (Belgium)	No	Recovered/resolving	Grapiprant
26 Oct 2023	GBR-ZOETISPV-2023-UK-03764	Bone and joint disorder	Zoetis (Belgium)	No	Unknown	Meloxicam
26 Oct 2023	ESP-ZOETISPV-2023-ES-00980	Joint pain; panting	Zoetis (Belgium)	No	Recovered/resolving	No

Date	Reference	Reaction	Sender	Serious	Outcome	Other medications
27 Oct 2023	FIN-ZOETISPV-2023-FI-00090	Musculoskeletal neoplasia; joint swelling	Zoetis (Belgium)	Yes	Recovered/resolving	Carprofen
27 Oct 2023	FIN-ZOETISPV-2023-FI-00096	Joint swelling	Zoetis (Belgium)	Yes	Fatal	Prednisolone
30 Oct 2023	CAN-ZOETISPV-2023-CA-04197	Lameness	Zoetis (Belgium)	No	Recovered/resolving	No
30 Oct 2023	FRA-ZOETISPV-2023-FR-00546	Polyarthritis	Zoetis (Belgium)	Yes	Euthanasia	No
31 Oct 2023	DEU-ZOETISPV-2023-DE-01197	Lameness; lack of efficacy	Zoetis (Belgium)	No	Recovered/resolving	Prednisolone
31 Oct 2023	GBR-ZOETISPV-2023-UK-03900	Swollen joint	Zoetis (Belgium)	No	Unknown	No
1 Nov 2023	POL-ZOETISPV-2023-PL-00157	Limb pitting oedema; joint pain	Zoetis (Belgium)	No	Recovered/resolving	No
6 Nov 2023	GBR-ZOETISPV-2023-UK-03877	Fracture; abnormal radiograph finding	Zoetis (Belgium)	Yes	Euthanasia	Meloxicam
6 Nov 2023	GBR-ZOETISPV-2023-UK-03879	Fracture; abnormal radiograph finding	Zoetis (Belgium)	Yes	Unknown	Meloxicam
6 Nov 2023	GBR-BIAHPV1P-23UK002270	Joint ligament disorder	Boehringer Ingelheim	No	Unknown	No
8 Nov 2023	USA-ZOETISPV-2023-US-39288	Lameness	Zoetis (Belgium)	No	Recovered/resolving	No
8 Nov 2023	ESP-ZOETISPV-2023-ES-01063	Chondrosarcoma; fracture	Zoetis (Belgium)	Yes	Recovered/resolving	No
8 Nov 2023	USA-ZOETISPV-2023-US-38637	Difficulty standing	Zoetis (Belgium)	Yes	Unknown	Adequan
9 Nov 2023	DEU-ZOETISPV-2023-DE-01253	Head down; restless	Zoetis (Belgium)	No	Recovered/resolving	No
13 Nov 2023	BEL-ZOETISPV-2023-BE-00192	Limping	Zoetis (Belgium)	No	Recovered/resolving	No
13 Nov 2023	USA-ZOETISPV-2023-US-40056	Limping	Zoetis (Belgium)	No	Recovered/resolving	No
13 Nov 2023	DNK-ZOETISPV-2023-DK-00106	Arthrosis	Zoetis (Belgium)	No	Unknown	Prednisolone
15 Nov 2023	AUS-ZOETISPV-2023-AU-01834	Head down; musculoskeletal pain	Zoetis (Belgium)	No	Recovered/resolving	No
15 Nov 2023	USA-ZOETISPV-2023-US-39314	Torn ligament	Zoetis (Belgium)	No	Recovered/resolving	Adequan
16 Nov 2023	DNK-ZOETISPV-2023-DK-00107	Polyarthritis	Zoetis (Belgium)	No	Recovered/resolving	Robenacoxib
20 Nov 2023	USA-ZOETISPV-2023-US-40629	Lameness and pain	Zoetis (Belgium)	No	Recovered/resolving	No
20 Nov 2023	ESP-ZOETISPV-2023-ES-01095	Fracture; abnormal test result; surgical site disorder	Zoetis (Belgium)	Yes	Recovered/resolving	No
22 Nov 2023	CZE-ZOETISPV-2023-CZ-00021	Lameness; reluctance to move	Zoetis (Belgium)	No	Unknown	No
22 Nov 2023	BEL-ZOETISPV-2023-BE-00208	Joint swelling	Zoetis (Belgium)	No	Recovered/resolving	Carprofen
23 Nov 2023	CAN-ZOETISPV-2023-CA-04618	Joint swelling, joint pain, abnormal radiograph finding	Zoetis (Belgium)	Yes	Recovered/resolving	No
23 Nov 2023	FRA-ZOETISPV-2023-FR-00641	Polyarthritis	Zoetis (Belgium)	Yes	Resolved with sequelae	No
24 Nov 2023	AUS-ZOETISPV-2023-AU-01879	Musculoskeletal neoplasia; arthritis; joint swelling	Zoetis (Belgium)	Yes	Euthanasia	No
27 Nov 2023	GBR-ZOETISPV-2023-UK-04279	Joint swelling, lameness	Zoetis (Belgium)	No	Unknown	No
27 Nov 2023	USA-ZOETISPV-2023-US-41452	Lameness	Zoetis (Belgium)	Yes	Fatal	No
27 Nov 2023	USA-ZOETISPV-2023-US-41927	Ligament rupture	Zoetis (Belgium)	No	Recovered/resolving	No

Date	Reference	Reaction	Sender	Serious	Outcome	Other medications
27 Nov 2023	USA-ZOETISPV-2023-US-41862	Difficulty standing; diarrhoea	Zoetis (Belgium)	No	Recovered/resolving	No
29 Nov 2023	USA-ZOETISPV-2023-US-42354	Non-weight bearing lameness	Zoetis (Belgium)	No	Unknown	No
29 Nov 2023	USA-ZOETISPV-2023-US-42391	Non-weight bearing lameness	Zoetis (Belgium)	No	Unknown	No
30 Nov 2023	USA-ZOETISPV-2023-US-42420	Joint effusion, joint pain	Zoetis (Belgium)	No	Ongoing	No
30 Nov 2023	USA-ZOETISPV-2023-US-42466	Ligament rupture	Zoetis (Belgium)	No	Ongoing	No
4 Dec 2023	DNK-ZOETISPV-2023-DK-00112	Ligament rupture; abnormal radiograph finding	Zoetis (Belgium)	No	Recovered/resolving	No
4 Dec 2023	USA-ZOETISPV-2023-US-42107	Joint pain	Zoetis (Belgium)	No	Ongoing	No
4 Dec 2023	USA-ZOETISPV-2023-US-42398	Ligament rupture	Zoetis (Belgium)	Yes	Ongoing	Grapiprant
4 Dec 2023	USA-ZOETISPV-2023-US-42546	Musculoskeletal pain	Zoetis (Belgium)	No	Ongoing	No
4 Dec 2023	USA-ZOETISPV-2023-US-43057	Lameness	Zoetis (Belgium)	No	Ongoing	Carprofen
5 Dec 2023	USA-ZOETISPV-2023-US-42725-1	Musculoskeletal disorder	Zoetis (Belgium)	No	Unknown	No
5 Dec 2023	USA-ZOETISPV-2023-US-42810	Musculoskeletal disorder	Zoetis (Belgium)	No	Unknown	No
5 Dec 2023	USA-ZOETISPV-2023-US-42725-2	Musculoskeletal disorder	Zoetis (Belgium)	No	Unknown	No
6 Dec 2023	USA-ZOETISPV-2023-US-43059	Osteosarcoma	Zoetis (Belgium)	Yes	Fatal	No
7 Dec 2023	USA-ZOETISPV-2023-US-43158	Joint pain	Zoetis (Belgium)	No	Unknown	No
8 Dec 2023	CAN-ZOETISPV-2023-CA-04917	Fracture; joint swelling; pain	Zoetis (Belgium)	Yes	Unknown	Meloxicam
11 Dec 2023	AUS-ZOETISPV-2023-AU-02104	Joint swelling; bone and joint disorder	Zoetis (Belgium)	No	Unknown	No
11 Dec 2023	FIN-ZOETISPV-2023-FI-00091	Musculoskeletal neoplasia; joint swelling	Zoetis (Belgium)	Yes	Recovered/resolving	Meloxicam
11 Dec 2023	USA-ZOETISPV-2023-US-42793	Difficulty going up and down stairs	Zoetis (Belgium)	No	Ongoing	No
12 Dec 2023	USA-ZOETISPV-2023-US-44269	Lameness	Zoetis (Belgium)	No	Ongoing	Lokivetmab, grapiprant
12 Dec 2023	USA-ZOETISPV-2023-US-44270	Non-weight bearing lameness	Zoetis (Belgium)	Yes	Fatal	Tramadol
13 Dec 2023	GBR-VMDDEFRA-02392/23	Joint swelling	Zoetis (Belgium)	No	Recovered/resolving	No
18 Dec 2023	USA-ZOETISPV-2023-US-43772	Pitting oedema, joint swelling	Zoetis (Belgium)	No	Ongoing	No
18 Dec 2023	USA-ZOETISPV-2023-US-44205	Non-weight bearing lameness, unable to stand	Zoetis (Belgium)	No	Ongoing	No
18 Dec 2023	USA-ZOETISPV-2023-US-44256	Musculoskeletal pain	Zoetis (Belgium)	No	Ongoing	No
18 Dec 2023	USA-ZOETISPV-2023-US-44436	Difficulty standing	Zoetis (Belgium)	No	Unknown	No
18 Dec 2023	AUS-ZOETISPV-2023-AU-02176	Musculoskeletal disorder; diarrhoea	Zoetis (Belgium)	No	Unknown	No
19 Dec 2023	USA-ZOETISPV-2023-US-45410	Non-weight bearing lameness	Zoetis (Belgium)	No	Ongoing	Ketamine
20 Dec 2023	DEU-ZOETISPV-2023-DE-01460	Swollen joint, bone and joint disorder	Zoetis (Belgium)	No	Unknown	No
20 Dec 2023	IRL-ZOETISPV-2022-IE-00088	Ligament rupture; limb collapse	Zoetis (Belgium)	Yes	Unknown	No

Date	Reference	Reaction	Sender	Serious	Outcome	Other medications
20 Dec 2023	BEL-ZOETISPV-2023-BE-00236 - 1	Arthrosis	Zoetis (Belgium)	No	Unknown	No
20 Dec 2023	BEL-ZOETISPV-2023-BE-00236 - 2	Arthrosis	Zoetis (Belgium)	No	Unknown	No
20 Dec 2023	BEL-ZOETISPV-2023-BE-00236 - 3	Arthrosis	Zoetis (Belgium)	No	Unknown	No
21 Dec 2023	NLD-ZOETISPV-2023-NL-00539	Lameness; partial lack of efficacy	Zoetis (Belgium)	No	Recovered/resolving	Prednisolone
21 Dec 2023	AUS-BIAHPV1P-23AU001262	Musculoskeletal neoplasia; joint swelling	Zoetis (Belgium)	Yes	Euthanasia	No
21 Dec 2023	USA-ZOETISPV-2023-US-45720	Lameness, difficulty getting up	Zoetis (Belgium)	Yes	Ongoing	No
25 Dec 2023	USA-ZOETISPV-2023-US-45275	Joint effusion	Zoetis (Belgium)	Yes	Ongoing	No
26 Dec 2023	USA-ZOETISPV-2023-US-45398	Collapse of leg, lameness	Zoetis (Belgium)	Yes	Recovered/normal	No
26 Dec 2023	USA-ZOETISPV-2023-US-46667	Joint swelling	Zoetis (Belgium)	No	Ongoing	No
26 Dec 2023	USA-ZOETISPV-2023-US-46821	Lameness	Zoetis (Belgium)	No	Ongoing	Grapiprant
27 Dec 2023	USA-ZOETISPV-2023-US-46824	Lameness	Zoetis (Belgium)	No	Ongoing	Grapiprant
27 Dec 2023	USA-ZOETISPV-2023-US-46825	Non-weight bearing lameness	Zoetis (Belgium)	No	Ongoing	No
1 Jan 2024	CAN-ZOETISPV-2023-CA-05236	Immune-mediated polyarthritis; septic arthritis	Zoetis (Belgium)	Yes	Recovered/resolving	No
1 Jan 2024	USA-ZOETISPV-2023-US-47324	Fracture	Zoetis (Belgium)	No	Unknown	No
4 Jan 2024	DEU-ZOETISPV-2023-DE-01575	Difficulty going up and down stairs; reluctant to move	Zoetis (Belgium)	No	Unknown	No
4 Jan 2024	USA-ZOETISPV-2023-US-47266	Lameness	Zoetis (Belgium)	Yes	Fatal	Meloxicam
5 Jan 2024	DEU-DEUPEIVM-2023-03014	Joint swelling, bone and joint disorder	Paul Ehrlich Institute	Yes	Recovered with sequelae	No
8 Jan 2024	CAN-ZOETISPV-2023-CA-05353	Lameness	Zoetis (Belgium)	No	Recovered/resolving	No
8 Jan 2024	USA-ZOETISPV-2023-US-48153	Musculoskeletal disorder	Zoetis (Belgium)	No	Unknown	No
8 Jan 2024	USA-ZOETISPV-2023-US-48232	Lameness	Zoetis (Belgium)	No	Ongoing	No
9 Jan 2024	CAN-ZOETISPV-2023-CA-05403	Ligament rupture	Zoetis (Belgium)	Yes	Ongoing	No
10 Jan 2024	CAN-ZOETISPV-2023-CA-05400	Limping; hyperkeratosis	Zoetis (Belgium)	No	Recovered/resolving	No
11 Jan 2024	CAN-ZOETISPV-2023-CA-05433	Limping, swollen feet	Zoetis (Belgium)	No	Recovered/normal	No
22 Jan 2024	AUS-ZOETISPV-2023-AU-02373	Bone and joint disorder (NOS); overdose	Zoetis (Belgium)	No	Unknown	No
23 Jan 2024	USA-ZOETISPV-2024-US-00525	Luxation/subluxation, torn ligament	Zoetis (Belgium)	No	Ongoing	No
23 Jan 2024	USA-ZOETISPV-2024-US-00991	Joint swelling	Zoetis (Belgium)	No	Ongoing	No
29 Jan 2024	AUT-ZOETISPV-2024-AT-00006	Lameness; muscle tremor	Zoetis (Belgium)	No	Recovered/resolving	No
29 Jan 2024	USA-ZOETISPV-2024-US-01280	Lameness	Zoetis (Belgium)	No	Ongoing	No
1 Feb 2024	USA-ZOETISPV-2024-US-02395	Swollen joint, joint pain	Zoetis (Belgium)	No	Ongoing	No
2 Feb 2024	DNK-DNKMEDAG-V20240008	Arthritis; lameness	Danish Medicines Agency	No	Recovered/resolving	No

Date	Reference	Reaction	Sender	Serious	Outcome	Other medications
5 Feb 2024	USA-ZOETISPV-2024-US-02150	Lameness	Zoetis (Belgium)	No	Ongoing	No
6 Feb 2024	GBR-ZOETISPV-2024-UK-00235	Joint swelling, bone and joint disorder	Zoetis (Belgium)	No	Ongoing	Meloxicam
6 Feb 2024	USA-ZOETISPV-2024-US-02104	Torn ligament	Zoetis (Belgium)	No	Ongoing	No
7 Feb 2024	CAN-ZOETISPV-2024-CA-00228	Limping, localised pain	Zoetis (Belgium)	Yes	Ongoing	No
7 Feb 2024	USA-ZOETISPV-2024-US-03268	Lameness	Zoetis (Belgium)	No	Ongoing	No
8 Feb 2024	GBR-ZOETISPV-2024-UK-00265	Lameness, abnormal radiograph finding	Zoetis (Belgium)	Yes	Fatal	Grapiprant
14 Feb 2024	GBR-ZOETISPV-2022-UK-00196	Joint effusion, lameness	Zoetis (Belgium)	No	Unknown	No
14 Feb 2024	USA-ZOETISPV-2024-US-03365	Ligament rupture	Zoetis (Belgium)	No	Ongoing	No
20 Feb 2024	USA-ZOETISPV-2024-US-04069	Musculoskeletal pain	Zoetis (Belgium)	No	Ongoing	No
22 Feb 2024	AUS-BIAHPV1P-24AU000248	Non-weight bearing lameness	Zoetis (Belgium)	No	Recovered/resolving	Butorphanol
26 Feb 2024	CHL-ZOETISPV-2024-CL-00025	Osteosarcoma	Zoetis (Belgium)	Yes	Unknown	No
26 Feb 2024	USA-ZOETISPV-2024-US-05158	Oedema of the extremities, fasciitis	Zoetis (Belgium)	No	Recovered/normal	No
27 Feb 2024	USA-ZOETISPV-2024-US-06053	Lameness	Zoetis (Belgium)	No	Recovered/normal	No
28 Feb 2024	USA-ZOETISPV-2024-US-05095	Swollen limb, limping	Zoetis (Belgium)	No	Ongoing	Grapiprant
1 Mar 2024	USA-ZOETISPV-2024-US-05220	Swollen joint, lameness	Zoetis (Belgium)	No	Ongoing	No
1 Mar 2024	USA-ZOETISPV-2024-US-05698	Joint effusion, lameness	Zoetis (Belgium)	No	Ongoing	No
4 Mar 2024	NLD-ZOETISPV-2024-NL-00060	Joint pain, lameness	Zoetis (Belgium)	No	Ongoing	No
4 Mar 2024	USA-ZOETISPV-2024-US-06435	Ligament rupture	Zoetis (Belgium)	No	Ongoing	No
13 Mar 2024	GBR-ZOETISPV-2024-UK-00900	Joint swelling	Zoetis (Belgium)	No	Unknown	No
13 Mar 2024	USA-ZOETISPV-2024-US-03313	Fracture	Zoetis (Belgium)	No	Recovered/normal	No
13 Mar 2024	USA-ZOETISPV-2024-US-07288	Lameness	Zoetis (Belgium)	No	Ongoing	No
18 Mar 2024	USA-ZOETISPV-2024-US-07949	Lameness	Zoetis (Belgium)	No	Ongoing	No
18 Mar 2024	USA-ZOETISPV-2024-US-08103	Limping	Zoetis (Belgium)	No	Recovered/normal	No
21 Mar 2024	USA-ZOETISPV-2024-US-08554	Swollen limb, difficulty standing	Zoetis (Belgium)	No	Unknown	No
21 Mar 2024	USA-ZOETISPV-2024-US-09658	Non-weight bearing lameness; abnormal radiograph	Zoetis (Belgium)	Yes	Ongoing	Prednisolone
25 Mar 2024	AUS-ZOETISPV-2024-AU-00451	Ligament rupture	Zoetis (Belgium)	No	Unknown	No
25 Mar 2024	AND-ZOETISPV-2024-AD-00003	Lameness; lack of efficacy	Zoetis (Belgium)	No	Unknown	No
25 Mar 2024	ESP-ZOETISPV-2024-ES-00208	Joint inflammation	Zoetis (Belgium)	No	Ongoing	No
26 Mar 2024	USA-ZOETISPV-2024-US-09184	Musculoskeletal disorder	Zoetis (Belgium)	No	Unknown	No
26 Mar 2024	GBR-ZOETISPV-2024-UK-00983	Lameness, bone and joint disorder	Zoetis (Belgium)	No	Unknown	No

Date	Reference	Reaction	Sender	Serious	Outcome	Other medications
26 Mar 2024	USA-ZOETISPV-2024-US-09515	Torn ligament	Zoetis (Belgium)	Yes	Ongoing	No
27 Mar 2024	USA-ZOETISPV-2024-US-09245 - 1	Torn ligament	Zoetis (Belgium)	No	Unknown	No
27 Mar 2024	USA-ZOETISPV-2024-US-09245 - 2	Torn ligament	Zoetis (Belgium)	No	Unknown	No
27 Mar 2024	USA-ZOETISPV-2024-US-09245 - 3	Torn ligament	Zoetis (Belgium)	No	Unknown	No
27 Mar 2024	USA-ZOETISPV-2024-US-09798	Luxation/subluxation, torn ligament	Zoetis (Belgium)	No	Unknown	No
28 Mar 2024	BEL-ZOETISPV-2024-BE-00059	Polyarthropathy	Zoetis (Belgium)	Yes	Unknown	Meloxicam
28 Mar 2024	AUS-F1A19E70-NLQBIOTICS_ST1751	Lameness; lack of efficacy	QBiotics (Netherlands)	No	Recovered/resolving	Tigilanol tiglate
28 Mar 2024	USA-ZOETISPV-2024-US-09650	Ligament rupture	Zoetis (Belgium)	No	Ongoing	Pentosan polysulphate
1 Apr 2024	GBR-VMDDEFRA-02229/23	Arthritis	VMD (Zoetis Belgium)	No	Recovered/resolving	Meloxicam
1 Apr 2024	GBR-ZOETISPV-2024-UK-01081	Joint swelling, arthritis	Zoetis (Belgium)	No	Unknown	Prednisolone
1 Apr 2024	USA-ZOETISPV-2024-US-11263	Lameness, abnormal radiograph finding	Zoetis (Belgium)	No	Unknown	No
4 Apr 2024	GBR-ZOETISPV-2024-UK-01044 - 1	Abnormal radiograph finding, Arthritis	Zoetis (Belgium)	No	Unknown	No
4 Apr 2024	GBR-ZOETISPV-2024-UK-01044 - 2	Abnormal radiograph finding, Arthritis	Zoetis (Belgium)	No	Unknown	No
4 Apr 2024	NLD-ZOETISPV-2024-NL-00124	Local swelling, reluctant to walk	Zoetis (Belgium)	No	Recovered/normal	No
4 Apr 2024	USA-ZOETISPV-2023-US-49512	Lameness	Zoetis (Belgium)	No	Unknown	No
4 Apr 2024	USA-ZOETISPV-2023-US-49515	Luxation/subluxation	Zoetis (Belgium)	No	Unknown	No
4 Apr 2024	USA-ZOETISPV-2024-US-13387	Ligament rupture	Zoetis (Belgium)	No	Unknown	No
4 Apr 2024	USA-ZOETISPV-2024-US-13430 - 1	Ligament disorder	Zoetis (Belgium)	No	Unknown	No
4 Apr 2024	USA-ZOETISPV-2024-US-13430 - 2	Ligament disorder	Zoetis (Belgium)	No	Unknown	No
4 Apr 2024	USA-ZOETISPV-2024-US-13430 - 3	Ligament disorder	Zoetis (Belgium)	No	Unknown	No
4 Apr 2024	USA-ZOETISPV-2024-US-13467	Limping	Zoetis (Belgium)	No	Unknown	No
4 Apr 2024	USA-ZOETISPV-2024-US-13529	Torn ligament	Zoetis (Belgium)	No	Unknown	No
5 Apr 2024	USA-ZOETISPV-2024-US-13205	Bone and joint disorder	Zoetis (Belgium)	No	Ongoing	No
8 Apr 2024	USA-ZOETISPV-2024-US-12048	Lameness, pain	Zoetis (Belgium)	No	Unknown	No
9 Apr 2024	DEU-ZOETISPV-2024-DE-00239	Joint swelling	Zoetis (Belgium)	No	Unknown	No
9 Apr 2024	USA-ZOETISPV-2024-US-11528	Musculoskeletal disorder	Zoetis (Belgium)	Yes	Unknown	No
11 Apr 2024	USA-ZOETISPV-2024-US-11996	Lameness	Zoetis (Belgium)	No	Unknown	No
11 Apr 2024	FRA-ZOETISPV-2024-FR-00207	Joint swelling, bone and joint disorder	Zoetis (Belgium)	No	Ongoing	No
12 Apr 2024	SWE-ZOETISPV-2024-SE-00048	Gait abnormality, localised pain	Zoetis (Belgium)	No	Ongoing	No
15 Apr 2024	USA-ZOETISPV-2024-US-12935	Musculoskeletal disorder	Zoetis (Belgium)	No	Ongoing	Grapiprant

Date	Reference	Reaction	Sender	Serious	Outcome	Other medications
16 Apr 2024	GBR-VMDDEFRA-00537/23	Arthritis	VMD (Zoetis Belgium)	No	Recovered/resolving	No
16 Apr 2024	GBR-VMDDEFRA-00552/23	Joint pain	Zoetis (Belgium)	No	Ongoing	No
16 Apr 2024	USA-ZOETISPV-2024-US-12502	Joint swelling	Zoetis (Belgium)	No	Ongoing	No
18 Apr 2024	DEU-ZOETISPV-2024-DE-00340	Swollen joint	Zoetis (Belgium)	Yes	Fatal	No
18 Apr 2024	USA-ZOETISPV-2024-US-03723	Limping, joint pain	Zoetis (Belgium)	No	Ongoing	No
19 Apr 2024	USA-ZOETISPV-2024-US-14184	Torn ligament	Zoetis (Belgium)	No	Ongoing	No
22 Apr 2024	USA-ZOETISPV-2024-US-13264	Joint swelling, lameness	Zoetis (Belgium)	No	Ongoing	Carprofen
22 Apr 2024	USA-ZOETISPV-2024-US-14020	Lameness, unable to walk	Zoetis (Belgium)	No	Recovered/normal	No
22 Apr 2024	USA-ZOETISPV-2024-US-14523	Limping	Zoetis (Belgium)	No	Ongoing	No
22 Apr 2024	USA-ZOETISPV-2024-US-14592	Joint effusion	Zoetis (Belgium)	No	Ongoing	No
25 Apr 2024	AUS-ZOETISPV-2024-AU-00672	Joint swelling; abnormal radiograph finding	Zoetis (Belgium)	No	Unknown	No
25 Apr 2024	AUS-ZOETISPV-2024-AU-00663	Septic arthritis	Zoetis (Belgium)	No	Recovered/resolving	No
25 Apr 2024	GBR-ZOETISPV-2024-UK-01549	Lameness, bone and joint disorder	Zoetis (Belgium)	No	Unknown	No
25 Apr 2024	USA-ZOETISPV-2023-US-49521	Musculoskeletal pain	Zoetis (Belgium)	No	Unknown	No
29 Apr 2024	CAN-ZOETISPV-2024-CA-01463	Limping	Zoetis (Belgium)	No	Recovered/resolving	No
29 Apr 2024	GBR-ZOETISPV-2024-UK-01552	Fracture, neoplasia	Zoetis (Belgium)	Yes	Recovered with sequelae	No
29 Apr 2024	USA-ZOETISPV-2024-US-14409	Lameness	Zoetis (Belgium)	No	Ongoing	No
30 Apr 2024	USA-ZOETISPV-2024-US-15020	Limping	Zoetis (Belgium)	No	Recovered/normal	No
3 May 2024	GBR-ZOETISPV-2024-UK-01670	Bone and joint disorder	Zoetis (Belgium)	Yes	Ongoing	Meloxicam
6 May 2024	USA-ZOETISPV-2024-US-15486	Musculoskeletal disorder	Zoetis (Belgium)	No	Unknown	No
6 May 2024	GBR-ZOETISPV-2024-UK-01828 - 1	Joint swelling	Zoetis (Belgium)	No	Unknown	No
6 May 2024	GBR-ZOETISPV-2024-UK-01828 - 2	Joint swelling	Zoetis (Belgium)	No	Unknown	No
6 May 2024	GBR-ZOETISPV-2024-UK-01828 - 3	Joint swelling	Zoetis (Belgium)	No	Unknown	No
6 May 2024	GBR-ZOETISPV-2024-UK-01828 - 4	Joint swelling	Zoetis (Belgium)	No	Unknown	No
6 May 2024	GBR-ZOETISPV-2024-UK-01831	Joint effusion, bone and joint disorder	Zoetis (Belgium)	No	Ongoing	Firocoxib
6 May 2024	SWE-ZOETISPV-2024-SE-00078	Arthritis	Zoetis (Belgium)	Yes	Fatal	No
6 May 2024	SWE-ZOETISPV-2024-SE-00079	Arthritis	Zoetis (Belgium)	No	Recovered with sequelae	No
6 May 2024	USA-ZOETISPV-2024-US-15864	Fracture	Zoetis (Belgium)	No	Ongoing	No
9 May 2024	USA-ZOETISPV-2024-US-16773	Cartilage degeneration	Zoetis (Belgium)	Yes	Ongoing	No
10 May 2024	GBR-VMDDEFRA-00194/24	Limb collapse; unable to stand	VMD (Zoetis Belgium)	Yes	Recovered/resolving	No

Date	Reference	Reaction	Sender	Serious	Outcome	Other medications
13 May 2024	NZL-ZOETISPV-2024-NZ-00118	Joint swelling, joint pain	Zoetis (Belgium)	No	Ongoing	No
13 May 2024	USA-ZOETISPV-2024-US-17005	Ligament disorder	Zoetis (Belgium)	No	Ongoing	No
13 May 2024	USA-ZOETISPV-2024-US-17290	Stiffness	Zoetis (Belgium)	No	Recovered/normal	No
14 May 2024	GBR-ZOETISPV-2024-UK-02016	Fracture	Zoetis (Belgium)	Yes	Unknown	No
14 May 2024	USA-ZOETISPV-2024-US-17256	Musculoskeletal disorder	Zoetis (Belgium)	No	Recovered/normal	No
16 May 2024	USA-ZOETISPV-2024-US-17789	Torn ligament	Zoetis (Belgium)	No	Ongoing	No
20 May 2024	DEU-ZOETISPV-2024-DE-00486	Limb collapse; difficulty standing	Zoetis (Belgium)	No	Recovered/resolving	No
20 May 2024	USA-ZOETISPV-2024-US-17958	Lameness	Zoetis (Belgium)	No	Recovered/normal	No
20 May 2024	USA-ZOETISPV-2024-US-18792	Non-weight bearing lameness	Zoetis (Belgium)	Yes	Ongoing	Carprofen
22 May 2024	GBR-ZOETISPV-2024-UK-01986	IMPA	Zoetis (Belgium)	Yes	Ongoing	No
22 May 2024	USA-ZOETISPV-2024-US-17724	Lameness	Zoetis (Belgium)	No	Ongoing	No
22 May 2024	XXI-ZOETISPV-2024-XI-00023	Non-weight bearing lameness	Zoetis (Belgium)	No	Unknown	No
23 May 2024	USA-ZOETISPV-2024-US-19067	Difficulty getting up, musculoskeletal disorder	Zoetis (Belgium)	No	Ongoing	Grapiprant
23 May 2024	USA-ZOETISPV-2024-US-19075	Joint swelling	Zoetis (Belgium)	No	Ongoing	No
29 May 2024	GBR-ZOETISPV-2024-UK-02231	Arthritis	Zoetis (Belgium)	No	Unknown	No
29 May 2024	GBR-BIAHPV1P-24UK001021	Ligament disorder	Boehringer Ingelheim	No	Unknown	Meloxicam
29 May 2024	USA-ZOETISPV-2024-US-19976	Fracture	Zoetis (Belgium)	No	Ongoing	No
30 May 2024	USA-ZOETISPV-2024-US-20973	Musculoskeletal disorder, pain	Zoetis (Belgium)	No	Unknown	No
30 May 2024	USA-ZOETISPV-2024-US-20110	Lameness	Zoetis (Belgium)	No	Recovered/normal	No
3 Jun 2024	GBR-ZOETISPV-2024-UK-02148	Limb swelling, non-weight bearing lameness	Zoetis (Belgium)	No	Ongoing	Robenacoxib
3 Jun 2024	USA-ZOETISPV-2024-US-20593	Joint swelling, arthritis	Zoetis (Belgium)	No	Ongoing	No
5 Jun 2024	GBR-VMDDEFRA-00490/24	Joint inflammation; autoimmune disorder	VMD (Zoetis Belgium)	No	Recovered/resolving	No
5 Jun 2024	AUS-ZOETISPV-2024-AU-00972	Hyperextension (plantigrade posture)	Zoetis (Belgium)	No	Unknown	No
5 Jun 2024	USA-ZOETISPV-2024-US-21643	Bone and joint disorder	Zoetis (Belgium)	Yes	Fatal	No
6 Jun 2024	USA-ZOETISPV-2024-US-21336	Non-weight bearing lameness; abnormal radiograph	Zoetis (Belgium)	No	Ongoing	No
7 Jun 2024	GBR-VMDDEFRA-00497/24	Bone and joint disorder (NOS); overdose	VMD (Zoetis Belgium)	No	Recovered/resolving	No
7 Jun 2024	GBR-DECHRALT-2024-08228	Lameness	Dechra Ltd	No	Unknown	No
10 Jun 2024	USA-ZOETISPV-2024-US-21928	Non-weight bearing lameness	Zoetis (Belgium)	No	Ongoing	No
11 Jun 2024	AUS-ZOETISPV-2024-AU-01001	Ligament rupture; hyperactivity	Zoetis (Belgium)	No	Recovered/resolving	No
11 Jun 2024	AUS-ZOETISPV-2024-AU-01002	Ligament rupture	Zoetis (Belgium)	Yes	Euthanasia	No

Date	Reference	Reaction	Sender	Serious	Outcome	Other medications
11 Jun 2024	USA-ZOETISPV-2024-US-22211	Fracture	Zoetis (Belgium)	Yes	Fatal	No
12 Jun 2024	USA-ZOETISPV-2024-US-06857	Ligament rupture	Zoetis (Belgium)	No	Unknown	No
13 Jun 2024	USA-ZOETISPV-2024-US-22583	Lameness	Zoetis (Belgium)	No	Ongoing	No
13 Jun 2024	USA-ZOETISPV-2024-US-22426	Joint cartilage disorder	Zoetis (Belgium)	No	Ongoing	No
18 Jun 2024	AUS-ZOETISPV-2024-AU-01049	Ligament rupture	Zoetis (Belgium)	No	Unknown	No
19 Jun 2024	USA-ZOETISPV-2024-US-23984	Arthritis	Zoetis (Belgium)	Yes	Fatal	Pentosan polysulphate
20 Jun 2024	USA-ZOETISPV-2024-US-23644	Lameness	Zoetis (Belgium)	No	Ongoing	No
21 Jun 2024	AUS-ZOETISPV-2023-AU-00952	Joint swelling	Zoetis (Belgium)	No	Unknown	No
24 Jun 2024	FRA-ZOETISPV-2024-FR-00416	Arthritis, abnormal radiograph finding	Zoetis (Belgium)	Yes	Fatal	No
24 Jun 2024	USA-ZOETISPV-2024-US-24008	Musculoskeletal disorder	Zoetis (Belgium)	No	Unknown	No
25 Jun 2024	CAN-ZOETISPV-2024-CA-00858	Arthritis, abnormal radiograph and cytology	Zoetis (Belgium)	Yes	Fatal	No
26 Jun 2024	USA-ZOETISPV-2024-US-24637	Joint effusion, abnormal radiograph finding	Zoetis (Belgium)	No	Ongoing	No
27 Jun 2024	USA-ZOETISPV-2024-US-24852	Lameness	Zoetis (Belgium)	No	Ongoing	No
27 Jun 2024	USA-ZOETISPV-2024-US-24895	Lameness, Musculoskeletal disorder	Zoetis (Belgium)	No	Ongoing	No
27 Jun 2024	USA-ZOETISPV-2024-US-24900	Arthritis, pain	Zoetis (Belgium)	No	Ongoing	No
28 Jun 2024	FIN-ZOETISPV-2024-FI-00117	Limping	Zoetis (Belgium)	No	Unknown	No
28 Jun 2024	USA-ZOETISPV-2024-US-14587	Joint effusion	Zoetis (Belgium)	No	Ongoing	No
1 Jul 2024	CAN-ZOETISPV-2024-CA-02609	Stiffness	Zoetis (Belgium)	No	Unknown	No
1 Jul 2024	CHE-ZOETISPV-2024-CH-00079	Lameness; reluctance to move; lack of efficacy	Zoetis (Belgium)	No	Resolved with sequelae	No
1 Jul 2024	AUS-ZOETISPV-2024-AU-00721	Joint swelling, joint pain	Zoetis (Belgium)	No	Unknown	Pentosan polysulphate
1 Jul 2024	BRA-ZOETISPV-2024-BR-02932	Joint oedema	Zoetis (Belgium)	No	Ongoing	No
1 Jul 2024	USA-ZOETISPV-2024-US-09412	Musculoskeletal disorder	Zoetis (Belgium)	No	Unknown	No
1 Jul 2024	USA-ZOETISPV-2024-US-26501	Musculoskeletal disorder	Zoetis (Belgium)	No	Recovered/normal	No
2 Jul 2024	GBR-VMDDEFRA-01032/24	Arthritis	VMD (Zoetis Belgium)	No	Recovered/resolving	No
2 Jul 2024	GBR-VMDDEFRA-01014/24	Fracture; arthritis	VMD (Zoetis Belgium)	No	Recovered/resolving	No
2 Jul 2024	GBR-VMDDEFRA-01033/24	Fracture; arthritis	VMD (Zoetis Belgium)	No	Recovered/resolving	No
2 Jul 2024	CHE-ZOETISPV-2024-CH-00035	Arthrosis	Zoetis (Belgium)	Yes	Unknown	No
2 Jul 2024	USA-ZOETISPV-2024-US-17710	Fracture	Zoetis (Belgium)	No	Ongoing	No
2 Jul 2024	USA-ZOETISPV-2024-US-25363	Torn ligament	Zoetis (Belgium)	No	Ongoing	No
2 Jul 2024	DNK-ZOETISPV-2024-DK-00071	Joint swelling, joint pain	Zoetis (Belgium)	Yes	Fatal	Lokivetmab

Date	Reference	Reaction	Sender	Serious	Outcome	Other medications
2 Jul 2024	USA-ZOETISPV-2024-US-25817	Joint swelling, abnormal radiograph finding	Zoetis (Belgium)	No	Ongoing	No
3 Jul 2024	GBR-VMDDEFRA-01034/24	Arthritis	VMD (Zoetis Belgium)	No	Recovered/resolving	No
3 Jul 2024	GBR-VMDDEFRA-01035/24	Arthritis	VMD (Zoetis Belgium)	No	Recovered/resolving	No
3 Jul 2024	USA-ZOETISPV-2024-US-26065	Lameness	Zoetis (Belgium)	No	Unknown	No
3 Jul 2024	USA-ZOETISPV-2024-US-25878	Joint swelling, abnormal radiograph finding	Zoetis (Belgium)	No	Ongoing	Carprofen
4 Jul 2024	CHE-ZOETISPV-2024-CH-00054	Immune-mediated polyarthritis; abnormal radiograph	Zoetis (Belgium)	Yes	Euthanasia	Firocoxib
4 Jul 2024	USA-ZOETISPV-2024-US-26085	Musculoskeletal disorder	Zoetis (Belgium)	No	Unknown	No
4 Jul 2024	USA-ZOETISPV-2024-US-26148	Non-weight bearing lameness	Zoetis (Belgium)	No	Ongoing	No
4 Jul 2024	USA-ZOETISPV-2024-US-26151	Ligament disorder	Zoetis (Belgium)	No	Unknown	No
4 Jul 2024	USA-ZOETISPV-2024-US-26153	Lameness, musculoskeletal pain	Zoetis (Belgium)	No	Ongoing	No
8 Jul 2024	AUS-ZOETISPV-2024-AU-01204	Musculoskeletal disorder; reluctant to move	Zoetis (Belgium)	No	Recovered/resolving	No
8 Jul 2024	USA-ZOETISPV-2024-US-26377	Musculoskeletal disorder	Zoetis (Belgium)	No	Unknown	No
8 Jul 2024	USA-ZOETISPV-2024-US-26260	Musculoskeletal disorder	Zoetis (Belgium)	No	Unknown	No
8 Jul 2024	GBR-ZOETISPV-2024-UK-02724	Arthritis, bone and joint disorder	Zoetis (Belgium)	No	Unknown	No
9 Jul 2024	USA-ZOETISPV-2024-US-26910	Musculoskeletal disorder	Zoetis (Belgium)	No	Unknown	No
9 Jul 2024	USA-ZOETISPV-2024-US-26767	Limping, reluctant to move	Zoetis (Belgium)	No	Ongoing	Lokivetmab
10 Jul 2024	ESP-ZOETISPV-2023-ES-00744	Osteosarcoma; fracture	Zoetis (Belgium)	Yes	Unknown	No
11 Jul 2024	USA-ZOETISPV-2024-US-27161	Bone and joint disorder	Zoetis (Belgium)	No	Ongoing	No
15 Jul 2024	USA-ZOETISPV-2024-US-27919	Musculoskeletal disorder	Zoetis (Belgium)	No	Unknown	No
16 Jul 2024	GBR-ZOETISPV-2024-UK-02905	Bone and joint disorder, overdose	Zoetis (Belgium)	No	Unknown	No
16 Jul 2024	USA-ZOETISPV-2024-US-27690	Swollen joint	Zoetis (Belgium)	No	Ongoing	No
18 Jul 2024	USA-ZOETISPV-2024-US-28474	Ligament disorder	Zoetis (Belgium)	No	Ongoing	Grapiprant
22 Jul 2024	USA-ZOETISPV-2024-US-28307	Lameness	Zoetis (Belgium)	No	Ongoing	No
22 Jul 2024	USA-ZOETISPV-2024-US-28598	Non-weight bearing lameness	Zoetis (Belgium)	Yes	Fatal	No
23 Jul 2024	BRA-ZOETISPV-2024-BR-03381	Limping	Zoetis (Belgium)	No	Unknown	No
23 Jul 2024	USA-ZOETISPV-2024-US-18583	Lameness, difficulty getting up	Zoetis (Belgium)	No	Ongoing	No
24 Jul 2024	GBR-ZOETISPV-2024-UK-02977	Fracture	Zoetis (Belgium)	No	Unknown	Meloxicam
26 Jul 2024	USA-ZOETISPV-2024-US-29243	Musculoskeletal disorder	Zoetis (Belgium)	No	Unknown	No
29 Jul 2024	BRA-ZOETISPV-2024-BR-03447	Non-weight bearing lameness	Zoetis (Belgium)	No	Unknown	No
29 Jul 2024	AUS-ZOETISPV-2024-AU-01301	Ligament disorder	Zoetis (Belgium)	Yes	Ongoing	Carprofen

Date	Reference	Reaction	Sender	Serious	Outcome	Other medications
29 Jul 2024	BRA-ZOETISPV-2024-BR-03447	Non-weight bearing lameness; reluctant to move	Zoetis (Belgium)	No	Unknown	No
29 Jul 2024	USA-ZOETISPV-2024-US-29340	Musculoskeletal disorder	Zoetis (Belgium)	Yes	Fatal	No
29 Jul 2024	USA-ZOETISPV-2024-US-29750	Torn ligament	Zoetis (Belgium)	No	Ongoing	No
30 Jul 2024	GBR-ZOETISPV-2024-UK-03071	Arthritis	Zoetis (Belgium)	Yes	Unknown	No
31 Jul 2024	USA-ZOETISPV-2024-US-29937	Joint swelling	Zoetis (Belgium)	No	Ongoing	No
1 Aug 2024	USA-ZOETISPV-2024-US-30542	Lameness, musculoskeletal pain	Zoetis (Belgium)	No	Unknown	No
1 Aug 2024	GBR-ZOETISPV-2024-UK-03140	Arthritis	Zoetis (Belgium)	No	Unknown	No
1 Aug 2024	GBR-ZOETISPV-2024-UK-03214	IMPA, luxation/subluxation	Zoetis (Belgium)	Yes	Ongoing	No
5 Aug 2024	AUS-ZOETISPV-2024-AU-01407	Joint swelling, bone and joint disorder	Zoetis (Belgium)	No	Unknown	No
5 Aug 2024	USA-ZOETISPV-2024-US-30333	Lameness	Zoetis (Belgium)	No	Unknown	No
5 Aug 2024	USA-ZOETISPV-2024-US-30554	Lameness	Zoetis (Belgium)	No	Recovered/normal	No
6 Aug 2024	GBR-ZOETISPV-2024-UK-03229	Bone and joint disorder, abnormal radiograph finding	Zoetis (Belgium)	Yes	Unknown	No
7 Aug 2024	BEL-ZOETISPV-2024-BE-00169	Osteosarcoma; joint swelling	Zoetis (Belgium)	Yes	Recovered/resolving	No
7 Aug 2024	GBR-ZOETISPV-2024-UK-03380	Septic arthritis	Zoetis (Belgium)	No	Unknown	No
8 Aug 2024	ESP-ZOETISPV-2024-ES-00718	Osteomyelitis	Zoetis (Belgium)	No	Unknown	Mavacoxib
9 Aug 2024	FRA-FRAANMVF-202404175	Arthrosis	French VMA	No	Recovered/resolving	No
9 Aug 2024	FRA-FRAANMVF-202404174	Joint pain, abnormal radiograph finding	NVMA France	No	Ongoing	No
9 Aug 2024	USA-ZOETISPV-2024-US-21986	Lameness	Zoetis (Belgium)	No	Ongoing	No
12 Aug 2024	CAN-ZOETISPV-2024-CA-03370	Lameness; stiffness; exercise intolerance	Zoetis (Belgium)	No	Unknown	No
13 Aug 2024	GBR-8BAF7B14-EQUIY-2024022	Joint pain; lameness	Zoetis (Belgium)	No	Recovered/resolving	Firocoxib; stem cells
13 Aug 2024	AUS-ZOETISPV-2024-AU-01313	Hyperextension (plantigrade posture)	Zoetis (Belgium)	No	Unknown	No
13 Aug 2024	USA-ZOETISPV-2024-US-32343	Limping, joint pain	Zoetis (Belgium)	No	Ongoing	Carprofen
13 Aug 2024	USA-ZOETISPV-2024-US-32346	Lameness and pain	Zoetis (Belgium)	No	Ongoing	Grapiprant
14 Aug 2024	USA-ZOETISPV-2024-US-32727	Non-weight bearing lameness; abnormal radiograph	Zoetis (Belgium)	Yes	Euthanasia	No
19 Aug 2024	GBR-ZOETISPV-2024-UK-03457	Joint swelling	Zoetis (Belgium)	No	Unknown	Robenacoxib
19 Aug 2024	GBR-ZOETISPV-2024-UK-03459	Joint swelling	Zoetis (Belgium)	No	Unknown	No
19 Aug 2024	GBR-ZOETISPV-2024-UK-03462	Joint swelling, bone and joint disorder	Zoetis (Belgium)	No	Unknown	No
19 Aug 2024	GBR-ZOETISPV-2024-UK-03549	Ligament rupture	Zoetis (Belgium)	No	Unknown	No
19 Aug 2024	USA-ZOETISPV-2024-US-32680	IMPA	Zoetis (Belgium)	Yes	Ongoing	No
19 Aug 2024	USA-ZOETISPV-2024-US-33237	Stiffness and pain	Zoetis (Belgium)	No	Recovered/normal	No

Date	Reference	Reaction	Sender	Serious	Outcome	Other medications
20 Aug 2024	SWE-SWEMEDAG-2024-00390	Lameness	Medical Products Agency	Yes	Ongoing	No
21 Aug 2024	GBR-ZOETISPV-2024-UK-03521	Ligament rupture	Zoetis (Belgium)	No	Unknown	Prednisolone
22 Aug 2024	AUS-ZOETISPV-2024-AU-01594	Musculoskeletal pain	Zoetis (Belgium)	No	Recovered/resolving	No
23 Aug 2024	GBR-VMDDEFRA-01318/24	Arthritis	VMD (Zoetis Belgium)	No	Recovered/resolving	No
23 Aug 2024	AUS-ZOETISPV-2024-AU-01571	Musculoskeletal disorder	Zoetis (Belgium)	No	Euthanasia	No
26 Aug 2024	DEU-ZOETISPV-2024-DE-01036	Gait abnormality	Zoetis (Belgium)	No	Recovered/resolving	No
26 Aug 2024	USA-ZOETISPV-2024-US-33761	Pitting oedema, non-weight bearing lameness	Zoetis (Belgium)	No	Ongoing	No
26 Aug 2024	USA-ZOETISPV-2024-US-33982	Lameness	Zoetis (Belgium)	No	Recovered/normal	No
27 Aug 2024	USA-ZOETISPV-2024-US-34032	Septic arthritis	Zoetis (Belgium)	No	Ongoing	Grapiprant
28 Aug 2024	GBR-VMDDEFRA-01442/24	Arthritis; abnormal histology (NOS); digit amputation	VMD (Zoetis Belgium)	Yes	Recovered/resolving	No
28 Aug 2024	USA-ZOETISPV-2024-US-33051	Musculoskeletal disorder	Zoetis (Belgium)	No	Ongoing	No
29 Aug 2024	GBR-ZOETISPV-2024-UK-03594	Joint swelling	Zoetis (Belgium)	No	Ongoing	No
29 Aug 2024	GBR-ZOETISPV-2024-UK-03599	IMPA	Zoetis (Belgium)	Yes	Unknown	Meloxicam
29 Aug 2024	USA-ZOETISPV-2024-US-34330	Osteosarcoma	Zoetis (Belgium)	Yes	Ongoing	No
30 Aug 2024	GBR-VMDDEFRA-01291/24	Fracture; lameness	VMD (Zoetis Belgium)	Yes	Recovered/resolving	No
2 Sep 2024	GBR-ZOETISPV-2024-UK-03644	Joint pain, bone and joint disorder	Zoetis (Belgium)	No	Ongoing	Firocoxib
2 Sep 2024	USA-ZOETISPV-2024-US-34732	Musculoskeletal disorder	Zoetis (Belgium)	Yes	Fatal	No
2 Sep 2024	USA-ZOETISPV-2024-US-34793	Torn ligament	Zoetis (Belgium)	No	Ongoing	No
2 Sep 2024	USA-ZOETISPV-2024-US-34833	Torn ligament	Zoetis (Belgium)	No	Ongoing	No
2 Sep 2024	USA-ZOETISPV-2024-US-34834	Torn ligament	Zoetis (Belgium)	No	Ongoing	No
4 Sep 2024	GBR-ZOETISPV-2024-UK-03732	Lameness, bone and joint disorder	Zoetis (Belgium)	No	Unknown	Meloxicam
5 Sep 2024	BEL-ZOETISPV-2024-BE-00107	Swollen joint, lameness	Zoetis (Belgium)	No	Recovered/normal	Cimicoxib
9 Sep 2024	DEU-ZOETISPV-2024-DE-01120	Tendinitis	Zoetis (Belgium)	No	Ongoing	No
9 Sep 2024	USA-ZOETISPV-2024-US-35982 - 1	Cartilage degeneration	Zoetis (Belgium)	No	Unknown	No
9 Sep 2024	USA-ZOETISPV-2024-US-35982 - 2	Cartilage degeneration	Zoetis (Belgium)	No	Unknown	No
9 Sep 2024	USA-ZOETISPV-2024-US-35982 - 3	Cartilage degeneration	Zoetis (Belgium)	No	Unknown	No
9 Sep 2024	USA-ZOETISPV-2024-US-36021	Lameness	Zoetis (Belgium)	No	Ongoing	No
9 Sep 2024	USA-ZOETISPV-2024-US-36276	Lameness, musculoskeletal pain	Zoetis (Belgium)	No	Ongoing	No
10 Sep 2024	CAN-ZOETISPV-2024-CA-03804	Lameness; difficulty going up and down stairs	Zoetis (Belgium)	No	Unknown	No
11 Sep 2024	FRA-ZOETISPV-2024-FR-00641	Arthritis; difficulty standing	Zoetis (Belgium)	No	Recovered/resolving	No

Date	Reference	Reaction	Sender	Serious	Outcome	Other medications
11 Sep 2024	USA-ZOETISPV-2024-US-36413	Musculoskeletal disorder	Zoetis (Belgium)	Yes	Ongoing	No
12 Sep 2024	IRL-IRLIMBVM-15/24	Arthritis, bone and joint disorder	HPRA	Yes	Unknown	No
19 Sep 2024	GBR-ZOETISPV-2024-UK-03542	Arthritis, joint swelling	Zoetis (Belgium)	No	Unknown	Grapiprant, meloxicam
19 Sep 2024	GBR-ZOETISPV-2024-UK-03921	Walking difficulty, head down	Zoetis (Belgium)	No	Unknown	No
19 Sep 2024	GBR-ZOETISPV-2022-UK-00127	Arthritis, joint swelling, lameness	Zoetis (Belgium)	Yes	Fatal	Grapiprant
19 Sep 2024	USA-ZOETISPV-2024-US-36364	Bone and joint disorder, abnormal radiograph finding	Zoetis (Belgium)	No	Unknown	No
19 Sep 2024	USA-ZOETISPV-2024-US-37469	Difficulty climbing stairs, unable to stand	Zoetis (Belgium)	No	Ongoing	No
19 Sep 2024	USA-ZOETISPV-2024-US-37504	Musculoskeletal disorder	Zoetis (Belgium)	No	Unknown	No
19 Sep 2024	USA-ZOETISPV-2024-US-37506	Torn ligament	Zoetis (Belgium)	No	Ongoing	Carprofen
20 Sep 2024	NZL-ZOETISPV-2024-NZ-00263	Swollen limb, pitting oedema, limb non-weight-bearing	Zoetis (Belgium)	Yes	Ongoing	Meloxicam
23 Sep 2024	AUS-ZOETISPV-2024-AU-01785	Bone and joint disorder, rigidity, stiffness	Zoetis (Belgium)	No	Recovered/normal	No
24 Sep 2024	GBR-ZOETISPV-2024-UK-04100	Bone and joint disorder, joint effusion	Zoetis (Belgium)	No	Unknown	No
24 Sep 2024	NLD-ZOETISPV-2024-NL-00471	Stiff gait	Zoetis (Belgium)	No	Recovered/normal	No
24 Sep 2024	USA-ZOETISPV-2024-US-37964	Torn ligament	Zoetis (Belgium)	No	Ongoing	No
24 Sep 2024	USA-ZOETISPV-2024-US-38824	Swollen joint, abnormal radiograph finding	Zoetis (Belgium)	No	Ongoing	No
25 Sep 2024	USA-ZOETISPV-2024-US-36335	Lameness, musculoskeletal pain	Zoetis (Belgium)	No	Ongoing	No
25 Sep 2024	USA-ZOETISPV-2024-US-38407	Lameness	Zoetis (Belgium)	No	Recovered/normal	No
25 Sep 2024	USA-ZOETISPV-2024-US-38495	Musculoskeletal disorder, unable to stand	Zoetis (Belgium)	No	Ongoing	No
25 Sep 2024	USA-ZOETISPV-2024-US-38397	Fracture	Zoetis (Belgium)	Yes	Fatal	No
26 Sep 2024	USA-ZOETISPV-2024-US-38424	Swollen joint	Zoetis (Belgium)	No	Ongoing	No
26 Sep 2024	USA-ZOETISPV-2024-US-38616	Lameness	Zoetis (Belgium)	No	Ongoing	Grapiprant
26 Sep 2024	USA-ZOETISPV-2024-US-38654	Osteosarcoma	Zoetis (Belgium)	Yes	Fatal	No
26 Sep 2024	USA-ZOETISPV-2024-US-39496	Limping	Zoetis (Belgium)	No	Ongoing	No
27 Sep 2024	USA-ZOETISPV-2024-US-37479	IMPA	Zoetis (Belgium)	Yes	Ongoing	No
30 Sep 2024	GBR-ZOETISPV-2024-UK-04142	Arthritis	Zoetis (Belgium)	No	Unknown	Firocoxib
30 Sep 2024	GBR-ZOETISPV-2024-UK-04143	Arthritis, abnormal test result	Zoetis (Belgium)	No	Unknown	Firocoxib
30 Sep 2024	GBR-ZOETISPV-2024-UK-04145	Bone and joint disorder	Zoetis (Belgium)	No	Unknown	No
30 Sep 2024	USA-ZOETISPV-2024-US-38728	Musculoskeletal disorder, unable to stand	Zoetis (Belgium)	No	Ongoing	No
30 Sep 2024	USA-ZOETISPV-2024-US-38851	Collapse of leg	Zoetis (Belgium)	No	Ongoing	No
30 Sep 2024	USA-ZOETISPV-2024-US-39312	Lameness, walking difficulty	Zoetis (Belgium)	No	Ongoing	No

Date	Reference	Reaction	Sender	Serious	Outcome	Other medications
30 Sep 2024	USA-ZOETISPV-2024-US-39362	Torn ligament	Zoetis (Belgium)	No	Recovered/normal	No
30 Sep 2024	USA-ZOETISPV-2024-US-39542	Gait abnormality	Zoetis (Belgium)	Yes	Ongoing	No
30 Sep 2024	GBR-ZOETISPV-2024-UK-04154	Joint swelling	Zoetis (Belgium)	No	Unknown	No
1 Oct 2024	USA-ZOETISPV-2024-US-39044	Swollen limb	Zoetis (Belgium)	No	Ongoing	No
1 Oct 2024	USA-ZOETISPV-2024-US-39275	Difficulty going up and down stairs	Zoetis (Belgium)	No	Recovered/normal	Carprofen
1 Oct 2024	AUS-ZOETISPV-2024-AU-01858	IMPA	Zoetis (Belgium)	Yes	Ongoing	Prednisolone
2 Oct 2024	GBR-ZOETISPV-2024-UK-04299	Fracture	Zoetis (Belgium)	Yes	Unknown	No
2 Oct 2024	GBR-ZOETISPV-2024-UK-04300	Fracture	Zoetis (Belgium)	Yes	Unknown	No
2 Oct 2024	GBR-ZOETISPV-2024-UK-04301	Bone and joint disorder	Zoetis (Belgium)	No	Unknown	No
2 Oct 2024	GBR-ZOETISPV-2024-UK-04302	Bone and joint disorder, cartilage disorder	Zoetis (Belgium)	Yes	Unknown	No
2 Oct 2024	GBR-ZOETISPV-2024-UK-04303	Bone and joint disorder	Zoetis (Belgium)	No	Unknown	No
2 Oct 2024	USA-ZOETISPV-2024-US-39299	Lameness, localised pain	Zoetis (Belgium)	No	Ongoing	No
2 Oct 2024	USA-ZOETISPV-2024-US-39443	Septic arthritis	Zoetis (Belgium)	Yes	Unknown	No
3 Oct 2024	BRA-ZOETISPV-2024-BR-04314	Joint pain, unable to stand	Zoetis (Belgium)	No	Recovered/normal	No
3 Oct 2024	BRA-ZOETISPV-2024-BR-04315	Joint pain, medication error	Zoetis (Belgium)	No	Recovered/normal	No
3 Oct 2024	USA-ZOETISPV-2024-US-39476	Pitting oedema, lameness	Zoetis (Belgium)	Yes	Ongoing	No
3 Oct 2024	USA-ZOETISPV-2024-US-40494	Musculoskeletal disorder	Zoetis (Belgium)	No	Unknown	No
7 Oct 2024	GBR-ZOETISPV-2024-UK-04255	Bone and joint disorder, lameness	Zoetis (Belgium)	No	Ongoing	No
7 Oct 2024	USA-ZOETISPV-2024-US-39731	Collapse of leg, joint swelling	Zoetis (Belgium)	No	Ongoing	Carprofen
7 Oct 2024	USA-ZOETISPV-2024-US-40102	Limping	Zoetis (Belgium)	Yes	Unknown	No
7 Oct 2024	USA-ZOETISPV-2024-US-40667	Bone and joint disorder, abnormal radiograph finding	Zoetis (Belgium)	Yes	Fatal	No
8 Oct 2024	FIN-ZOETISPV-2024-FI-00240	Head down	Zoetis (Belgium)	No	Unknown	No
8 Oct 2024	USA-ZOETISPV-2024-US-40230	Collapse of leg	Zoetis (Belgium)	No	Recovered/normal	No
8 Oct 2024	USA-ZOETISPV-2024-US-40430	Joint oedema, musculoskeletal disorder	Zoetis (Belgium)	No	Ongoing	Carprofen
8 Oct 2024	USA-ZOETISPV-2024-US-40609	Musculoskeletal disorder	Zoetis (Belgium)	No	Ongoing	No
8 Oct 2024	USA-ZOETISPV-2024-US-40671	Torn ligament	Zoetis (Belgium)	Yes	Unknown	No
9 Oct 2024	USA-ZOETISPV-2024-US-39716	Joint swelling, bone and joint disorder	Zoetis (Belgium)	No	Ongoing	No
10 Oct 2024	ESP-ZOETISPV-2024-ES-00910	Lameness	Zoetis (Belgium)	No	Recovered/normal	No
10 Oct 2024	GBR-ZOETISPV-2023-UK-04868	Fracture, overdose	Zoetis (Belgium)	Yes	Unknown	Robenacoxib
10 Oct 2024	USA-ZOETISPV-2024-US-40524	Joint effusion, non-weight bearing lameness	Zoetis (Belgium)	No	Ongoing	No

Date	Reference	Reaction	Sender	Serious	Outcome	Other medications
14 Oct 2024	USA-ZOETISPV-2024-US-38729	Collapse of leg	Zoetis (Belgium)	No	Recovered/normal	Grapiprant
14 Oct 2024	USA-ZOETISPV-2024-US-41199	Difficulty going up and down stairs	Zoetis (Belgium)	No	Unknown	No
14 Oct 2024	USA-ZOETISPV-2024-US-41241	Joint effusion	Zoetis (Belgium)	No	Unknown	No
14 Oct 2024	USA-ZOETISPV-2024-US-41293	Lameness	Zoetis (Belgium)	No	Ongoing	Grapiprant
15 Oct 2024	GBR-ZOETISPV-2024-UK-04427	Joint swelling, abnormal radiograph finding	Zoetis (Belgium)	Yes	Ongoing	No
15 Oct 2024	USA-ZOETISPV-2024-US-41854	Musculoskeletal disorder	Zoetis (Belgium)	No	Ongoing	No
15 Oct 2024	USA-ZOETISPV-2023-US-47818	Polymyositis	Zoetis (Belgium)	Yes	Ongoing	No
16 Oct 2024	FRA-FRAANMVF-202405794	Musculoskeletal disorder, stiff limb	NVMA France	No	Unknown	No
16 Oct 2024	USA-ZOETISPV-2024-US-20228	Musculoskeletal disorder, unable to walk	Zoetis (Belgium)	No	Unknown	No
16 Oct 2024	USA-ZOETISPV-2024-US-41336	Musculoskeletal disorder, unable to stand	Zoetis (Belgium)	No	Recovered/normal	No
16 Oct 2024	USA-ZOETISPV-2024-US-41738	Swollen joint, unable to rise	Zoetis (Belgium)	No	Ongoing	No
16 Oct 2024	USA-ZOETISPV-2024-US-41861	Ligament disorder, luxation/subluxation	Zoetis (Belgium)	No	Ongoing	No
17 Oct 2024	CAN-ZOETISPV-2024-CA-04490	IMPA	Zoetis (Belgium)	Yes	Ongoing	Meloxicam
17 Oct 2024	ESP-DECHRALT-2024-15597	Lameness	Dechra Ltd	No	Recovered/normal	No
17 Oct 2024	GBR-ZOETISPV-2024-UK-04379	Joint effusion, abnormal radiograph finding	Zoetis (Belgium)	Yes	Unknown	No
17 Oct 2024	GBR-ZOETISPV-2024-UK-04751	Fracture, abnormal radiograph finding	Zoetis (Belgium)	Yes	Unknown	Firocoxib
17 Oct 2024	GBR-ZOETISPV-2024-UK-04758	Bone and joint disorder	Zoetis (Belgium)	No	Unknown	Lokivetmab
17 Oct 2024	USA-ZOETISPV-2024-US-41708	Musculoskeletal disorder	Zoetis (Belgium)	No	Unknown	No
17 Oct 2024	USA-ZOETISPV-2024-US-41732	Musculoskeletal disorder	Zoetis (Belgium)	No	Unknown	No
17 Oct 2024	USA-ZOETISPV-2024-US-41755	Difficulty going up and down stairs	Zoetis (Belgium)	No	Unknown	No
18 Oct 2024	GBR-ZOETISPV-2024-UK-03698	Bone and joint disorder, abnormal radiograph finding	Zoetis (Belgium)	No	Unknown	Robenacoxib
18 Oct 2024	GBR-ZOETISPV-2024-UK-03722	Bone and joint disorder	Zoetis (Belgium)	No	Unknown	No
21 Oct 2024	GBR-ZOETISPV-2024-UK-04770	Joint effusion, bone and joint disorder	Zoetis (Belgium)	No	Unknown	Robenacoxib
21 Oct 2024	USA-ZOETISPV-2024-US-41687	Musculoskeletal disorder	Zoetis (Belgium)	No	Unknown	No
21 Oct 2024	USA-ZOETISPV-2024-US-42349	Luxation/subluxation, joint swelling	Zoetis (Belgium)	No	Ongoing	No
21 Oct 2024	USA-ZOETISPV-2024-US-42411	Bone and joint disorder	Zoetis (Belgium)	No	Ongoing	Firocoxib
22 Oct 2024	GBR-ZOETISPV-2024-UK-03883	Bone and joint disorder, synovitis	Zoetis (Belgium)	Yes	Unknown	Carprofen
22 Oct 2024	USA-ZOETISPV-2024-US-35154	Musculoskeletal disorder	Zoetis (Belgium)	Yes	Fatal	No
22 Oct 2024	USA-ZOETISPV-2024-US-41812	Bone and joint disorder, cartilage degeneration	Zoetis (Belgium)	No	Unknown	Firocoxib
23 Oct 2024	CAN-ZOETISPV-2024-CA-04575	Osteomyelitis	Zoetis (Belgium)	Yes	Ongoing	No

Date	Reference	Reaction	Sender	Serious	Outcome	Other medications
23 Oct 2024	USA-ZOETISPV-2024-US-42334	Ligament rupture	Zoetis (Belgium)	No	Ongoing	No
24 Oct 2024	ESP-ZOETISPV-2024-ES-00956	Joint inflammation	Zoetis (Belgium)	Yes	Fatal	Lokivetmab
24 Oct 2024	IRL-ZOETISPV-2024-IE-00212	Lameness	Zoetis (Belgium)	No	Ongoing	No
24 Oct 2024	FRA-ZOETISPV-2022-FR-00722	Joint pain	Zoetis (Belgium)	No	Recovered/normal	No
25 Oct 2024	GBR-VMDDEFRA-01939/24	Ligament disorder, abnormal radiograph finding	Zoetis (Belgium)	No	Ongoing	No
26 Oct 2024	DEU-DEUPEIVM-2023-02265	Joint swelling, abnormal radiograph finding	Paul Ehrlich Institute	Yes	Recovered/resolving	No
28 Oct 2024	DEU-ZOETISPV-2024-DE-01374	Bone and joint disorder	Zoetis (Belgium)	No	Unknown	No
28 Oct 2024	USA-ZOETISPV-2024-US-43269	Lameness	Zoetis (Belgium)	Yes	Fatal	No
28 Oct 2024	USA-ZOETISPV-2024-US-43305	Joint inflammation, bone and joint disorder	Zoetis (Belgium)	No	Ongoing	Carprofen
28 Oct 2024	USA-ZOETISPV-2024-US-43590	Limping	Zoetis (Belgium)	No	Unknown	No
28 Oct 2024	USA-ZOETISPV-2024-US-44266	Limping, musculoskeletal pain	Zoetis (Belgium)	No	Ongoing	Grapiprant
29 Oct 2024	CAN-ZOETISPV-2024-CA-04677	Lameness	Zoetis (Belgium)	No	Recovered/normal	No
29 Oct 2024	NOR-ZOETISPV-2024-NO-00178	Joint oedema	Zoetis (Belgium)	No	Unknown	No
29 Oct 2024	USA-ZOETISPV-2024-US-43556	Plantigrade posture, joint swelling	Zoetis (Belgium)	No	Ongoing	Grapiprant
29 Oct 2024	USA-ZOETISPV-2024-US-43980	Joint swelling	Zoetis (Belgium)	No	Ongoing	No
30 Oct 2024	USA-F1A19E70-NLQBIOTICS_ST2056	Swollen limb, unable to walk	QBiotics (NL)	No	Unknown	No
31 Oct 2024	USA-ZOETISPV-2024-US-39746	Musculoskeletal disorder	Zoetis (Belgium)	Yes	Ongoing	No
4 Nov 2024	AUS-ZOETISPV-2024-AU-02119	Fracture, ligament disorder, cellulitis	Zoetis (Belgium)	Yes	Unknown	Firocoxib
4 Nov 2024	CAN-ZOETISPV-2024-CA-04739	Luxation/subluxation	Zoetis (Belgium)	No	Ongoing	Robenacoxib
4 Nov 2024	GBR-ZOETISPV-2024-UK-04726	Abnormal radiograph finding, arthritis	Zoetis (Belgium)	Yes	Unknown	No
4 Nov 2024	GBR-ZOETISPV-2024-UK-04746	Joint effusion	Zoetis (Belgium)	Yes	Unknown	Robenacoxib
4 Nov 2024	GBR-ZOETISPV-2024-UK-04749	Joint effusion	Zoetis (Belgium)	No	Unknown	Meloxicam
4 Nov 2024	GBR-ZOETISPV-2024-UK-04752	Joint effusion, bone and joint disorder	Zoetis (Belgium)	No	Unknown	No
4 Nov 2024	GBR-ZOETISPV-2024-UK-04753	Joint swelling, bone and joint disorder	Zoetis (Belgium)	Yes	Fatal	Meloxicam, robenacoxib
4 Nov 2024	GBR-ZOETISPV-2024-UK-04757	Fracture, abnormal radiograph finding, uncoded sign	Zoetis (Belgium)	Yes	Unknown	No
4 Nov 2024	GBR-ZOETISPV-2024-UK-04761	Joint effusion, abnormal radiograph finding	Zoetis (Belgium)	No	Unknown	Robenacoxib
4 Nov 2024	GBR-ZOETISPV-2024-UK-04765	Septic arthritis	Zoetis (Belgium)	No	Unknown	No
4 Nov 2024	GBR-ZOETISPV-2024-UK-04769	Fracture	Zoetis (Belgium)	Yes	Unknown	Carprofen
4 Nov 2024	GBR-ZOETISPV-2024-UK-04783	Limping	Zoetis (Belgium)	No	Unknown	No
4 Nov 2024	USA-ZOETISPV-2024-US-44054	Collapse of leg	Zoetis (Belgium)	No	Unknown	No

Date	Reference	Reaction	Sender	Serious	Outcome	Other medications
4 Nov 2024	USA-ZOETISPV-2024-US-44122	Difficulty rising	Zoetis (Belgium)	No	Recovered/normal	No
4 Nov 2024	USA-ZOETISPV-2024-US-44220	Musculoskeletal disorder, pain, overdose	Zoetis (Belgium)	No	Ongoing	No
4 Nov 2024	USA-ZOETISPV-2024-US-44916	Swollen limb, non-weight bearing lameness	Zoetis (Belgium)	Yes	Ongoing	No
5 Nov 2024	ITA-ZOETISPV-2024-IT-00267	Bone and joint disorder	Zoetis (Belgium)	No	Unknown	No
5 Nov 2024	ITA-ZOETISPV-2024-IT-00268	Bone and joint disorder	Zoetis (Belgium)	No	Unknown	No
5 Nov 2024	USA-ZOETISPV-2024-US-44387	Fracture, neoplasia	Zoetis (Belgium)	Yes	Fatal	No
5 Nov 2024	USA-ZOETISPV-2024-US-44629	Swollen joint, bone and joint disorder	Zoetis (Belgium)	No	Ongoing	Carprofen
5 Nov 2024	USA-ZOETISPV-2024-US-44770	Swollen joint, bone and joint disorder	Zoetis (Belgium)	No	Ongoing	Carprofen
6 Nov 2024	USA-ZOETISPV-2024-US-44536	Ligament rupture, luxation/subluxation	Zoetis (Belgium)	No	Ongoing	No
6 Nov 2024	USA-ZOETISPV-2024-US-44575	Limping, localised oedema	Zoetis (Belgium)	No	Ongoing	No
6 Nov 2024	USA-ZOETISPV-2024-US-44832	Limping	Zoetis (Belgium)	Yes	Fatal	Grapiprant
7 Nov 2024	AUS-ZOETISPV-2024-AU-01800	Arthritis, lameness	Zoetis (Belgium)	No	Ongoing	No
7 Nov 2024	GBR-ZOETISPV-2024-UK-04864	Fracture, abnormal histology	Zoetis (Belgium)	Yes	Recovered/normal	No
7 Nov 2024	GBR-ZOETISPV-2024-UK-04891	Joint effusion, abnormal radiograph finding, uncoded sign	Zoetis (Belgium)	No	Unknown	No
7 Nov 2024	GRC-ZOETISPV-2024-GR-00079	Joint swelling	Zoetis (Belgium)	No	Recovered/normal	No
7 Nov 2024	GRC-ZOETISPV-2024-GR-00080	Joint swelling	Zoetis (Belgium)	No	Recovered/normal	No
7 Nov 2024	USA-ZOETISPV-2024-US-19641	Collapse of leg	Zoetis (Belgium)	No	Ongoing	No
8 Nov 2024	USA-ZZELANCO-US2024_052775	Arthritis	Elanco	No	Ongoing	No
11 Nov 2024	AUS-ZOETISPV-2024-AU-02179	Collapse of leg	Zoetis (Belgium)	Yes	Unknown	No
11 Nov 2024	GBR-ZOETISPV-2024-UK-04854	IMPA	Zoetis (Belgium)	Yes	Ongoing	No
11 Nov 2024	USA-ZOETISPV-2024-US-44735	Fracture, neoplasia	Zoetis (Belgium)	Yes	Fatal	No
11 Nov 2024	USA-ZOETISPV-2024-US-44983	Ligament disorder	Zoetis (Belgium)	No	Ongoing	No
11 Nov 2024	USA-ZOETISPV-2024-US-45204	Lameness	Zoetis (Belgium)	No	Ongoing	No
11 Nov 2024	USA-ZOETISPV-2024-US-45401	Musculoskeletal disorder, unable to stand	Zoetis (Belgium)	No	Recovered/normal	No
12 Nov 2024	GBR-ZOETISPV-2024-UK-04941	Septic arthritis, abnormal radiograph finding	Zoetis (Belgium)	Yes	Unknown	No
12 Nov 2024	GBR-ZOETISPV-2024-UK-04943	Swollen joint, abnormal radiograph finding	Zoetis (Belgium)	Yes	Unknown	No
12 Nov 2024	LUX-ZOETISPV-2024-BE-00244	Joint swelling, synovitis	Zoetis (Belgium)	No	Ongoing	No
12 Nov 2024	USA-ZOETISPV-2024-US-46145	Limping	Zoetis (Belgium)	No	Recovered/normal	No
13 Nov 2024	CAN-DECHRALT-2024-01512	Arthritis	Vetcare Oy	Yes	Fatal	Rimadyl
13 Nov 2024	GBR-ZOETISPV-2024-UK-04935	Joint swelling, uncoded sign	Zoetis (Belgium)	No	Ongoing	No

Date	Reference	Reaction	Sender	Serious	Outcome	Other medications
13 Nov 2024	USA-ZOETISPV-2024-US-45844	Collapse of leg	Zoetis (Belgium)	No	Ongoing	No
13 Nov 2024	USA-ZOETISPV-2024-US-45891	Non-weight bearing lameness	Zoetis (Belgium)	No	Recovered/normal	Lokivetmab
13 Nov 2024	USA-ZOETISPV-2024-US-45897	Musculoskeletal disorder	Zoetis (Belgium)	No	Unknown	No
14 Nov 2024	GBR-VMDDEFRA-02119/24	Fracture	Zoetis (Belgium)	No	Ongoing	No
14 Nov 2024	GBR-ZOETISPV-2024-UK-04900	Fracture, abnormal radiograph finding, IMPA	Zoetis (Belgium)	Yes	Unknown	Meloxicam
14 Nov 2024	USA-ZOETISPV-2024-US-45703	Musculoskeletal disorder	Zoetis (Belgium)	Yes	Fatal	No
15 Nov 2024	GBR-VMDDEFRA-02182/24	Arthritis	Zoetis (Belgium)	No	Ongoing	No
18 Nov 2024	GBR-ZOETISPV-2024-UK-05007	Joint swelling, abnormal posture	Zoetis (Belgium)	No	Unknown	Meloxicam
18 Nov 2024	GBR-ZOETISPV-2024-UK-05009	Septic arthritis	Zoetis (Belgium)	No	Unknown	No
18 Nov 2024	GBR-ZOETISPV-2024-UK-05011	Joint swelling	Zoetis (Belgium)	No	Unknown	No
18 Nov 2024	USA-ZOETISPV-2024-US-46217	Joint swelling, joint pain, abnormal cytology	Zoetis (Belgium)	No	Ongoing	No
18 Nov 2024	USA-ZOETISPV-2024-US-46257	Joint swelling, joint pain, abnormal radiograph finding	Zoetis (Belgium)	No	Ongoing	Lokivetmab
18 Nov 2024	USA-ZOETISPV-2024-US-46281	Luxation/subluxation, joint swelling	Zoetis (Belgium)	No	Ongoing	No
18 Nov 2024	USA-ZOETISPV-2024-US-46432	Ligament rupture, luxation/subluxation	Zoetis (Belgium)	No	Ongoing	No
18 Nov 2024	USA-ZOETISPV-2024-US-46481	Joint swelling, lameness	Zoetis (Belgium)	No	Unknown	Carprofen
18 Nov 2024	USA-ZOETISPV-2024-US-46584	Non-weight bearing lameness	Zoetis (Belgium)	No	Ongoing	No
19 Nov 2024	USA-ZOETISPV-2024-US-46687	Bone and joint disorder, abnormal histology	Zoetis (Belgium)	Yes	Ongoing	Carprofen
19 Nov 2024	USA-ZOETISPV-2024-US-46724	Torn ligament	Zoetis (Belgium)	No	Ongoing	No
19 Nov 2024	USA-ZOETISPV-2024-US-46877	Arthritis	Zoetis (Belgium)	No	Unknown	No
19 Nov 2024	USA-ZOETISPV-2024-US-46878	Arthritis	Zoetis (Belgium)	No	Unknown	No
20 Nov 2024	AUS-ZOETISPV-2024-AU-02273	Lameness	Zoetis (Belgium)	No	Unknown	No
20 Nov 2024	USA-ZOETISPV-2024-US-42690	Ligament rupture	Zoetis (Belgium)	Yes	Unknown	No
20 Nov 2024	USA-ZOETISPV-2024-US-46729	Joint effusion, bone and joint disorder	Zoetis (Belgium)	No	Ongoing	Grapiprant
21 Nov 2024	AUS-ZOETISPV-2024-AU-02054	IMPA	Zoetis (Belgium)	Yes	Ongoing	Prednisolone
21 Nov 2024	GBR-ZOETISPV-2024-UK-04376	Joint swelling	Zoetis (Belgium)	No	Unknown	Firocoxib
21 Nov 2024	USA-ZOETISPV-2024-US-47048	Joint swelling, limping	Zoetis (Belgium)	No	Ongoing	No
22 Nov 2024	AUS-ZOETISPV-2024-AU-02283	Joint swelling, lameness	Zoetis (Belgium)	Yes	Unknown	No
25 Nov 2024	CAN-ZOETISPV-2024-CA-05086	Bone and joint disorder, movement of implant	Zoetis (Belgium)	Yes	Recovered with sequelae	No
25 Nov 2024	GBR-ZOETISPV-2024-UK-05102	Arthritis	Zoetis (Belgium)	No	Unknown	No
25 Nov 2024	USA-ZOETISPV-2024-US-47124	Lameness, localised pain	Zoetis (Belgium)	No	Ongoing	No

Date	Reference	Reaction	Sender	Serious	Outcome	Other medications
25 Nov 2024	USA-ZOETISPV-2024-US-47134	Musculoskeletal disorder, difficulty walking	Zoetis (Belgium)	No	Unknown	No
25 Nov 2024	USA-ZOETISPV-2024-US-47522	Luxation/subluxation, joint effusion	Zoetis (Belgium)	No	Ongoing	Grapiprant
25 Nov 2024	USA-ZOETISPV-2024-US-48067	Joint swelling, limping	Zoetis (Belgium)	No	Ongoing	Grapiprant
25 Nov 2024	USA-ZOETISPV-2024-US-48070	IMPA	Zoetis (Belgium)	Yes	Ongoing	No
26 Nov 2024	CAN-ZOETISPV-2024-CA-05085	Fracture, abnormal radiograph finding, abnormal histology	Zoetis (Belgium)	Yes	Recovered with sequelae	Meloxicam
26 Nov 2024	DEU-ZOETISPV-2024-DE-01522	Gait abnormality. stiffness	Zoetis (Belgium)	Yes	Ongoing	Grapiprant
26 Nov 2024	GBR-ZOETISPV-2024-UK-05114	Gait abnormality	Zoetis (Belgium)	No	Unknown	No
26 Nov 2024	GBR-ZOETISPV-2024-UK-05124	Joint swelling, joint pain	Zoetis (Belgium)	No	Unknown	No
26 Nov 2024	USA-ZOETISPV-2024-US-47407	Joint swelling, joint pain, bone and joint disorder	Zoetis (Belgium)	No	Ongoing	No
26 Nov 2024	USA-ZOETISPV-2024-US-47409	Bone and joint disorder, difficulty standing	Zoetis (Belgium)	No	Ongoing	No
27 Nov 2024	CAN-ZOETISPV-2024-CA-05148	Abnormal radiograph finding, arthritis, uncoded sign	Zoetis (Belgium)	No	Unknown	No
28 Nov 2024	USA-ZOETISPV-2024-US-48400	Limping, walking difficulty	Zoetis (Belgium)	No	Unknown	No
2 Dec 2024	CAN-ZOETISPV-2024-CA-05182	Difficulty climbing stairs, stiffness	Zoetis (Belgium)	No	Ongoing	No
2 Dec 2024	USA-BIAHPV1P-24US049653	Arthritis	Boehringer Ingelheim	No	Ongoing	No
2 Dec 2024	USA-ZOETISPV-2024-US-48143	Collapse of leg	Zoetis (Belgium)	No	Ongoing	No
2 Dec 2024	USA-ZOETISPV-2024-US-48527	Bone and joint disorder, abnormal radiograph finding	Zoetis (Belgium)	No	Ongoing	Carprofen
3 Dec 2024	USA-ZOETISPV-2024-US-48701	Luxation/subluxation	Zoetis (Belgium)	No	Unknown	No
3 Dec 2024	USA-ZOETISPV-2024-US-48829	Plantigrade posture, joint pain	Zoetis (Belgium)	No	Ongoing	Carprofen
4 Dec 2024	USA-ZOETISPV-2024-US-40657	Septic arthritis	Zoetis (Belgium)	Yes	Fatal	No
4 Dec 2024	USA-ZOETISPV-2024-US-48835	Limping	Zoetis (Belgium)	No	Recovered/normal	No
5 Dec 2024	ESP-ZOETISPV-2024-ES-01094	Bone and joint disorder	Zoetis (Belgium)	Yes	Fatal	No
5 Dec 2024	GBR-ZOETISPV-2024-UK-05310	Joint swelling, bone and joint disorder	Zoetis (Belgium)	No	Unknown	No
5 Dec 2024	GBR-ZOETISPV-2024-UK-05346	Swollen joint, bone and joint disorder	Zoetis (Belgium)	Yes	Unknown	No
5 Dec 2024	USA-ZOETISPV-2024-US-49167	IMPA	Zoetis (Belgium)	Yes	Ongoing	Tramadol
5 Dec 2024	USA-ZOETISPV-2024-US-49294	Ligament disorder, unable to walk	Zoetis (Belgium)	No	Ongoing	No
5 Dec 2024	USA-ZOETISPV-2024-US-50038	Ligament rupture, abnormal radiograph finding	Zoetis (Belgium)	No	Ongoing	No
5 Dec 2024	USA-ZOETISPV-2024-US-50050	Collapse of leg	Zoetis (Belgium)	No	Unknown	No
9 Dec 2024	AUS-ZOETISPV-2024-AU-02399	Ligament rupture, abnormal radiograph finding	Zoetis (Belgium)	Yes	Unknown	No
9 Dec 2024	CAN-ZOETISPV-2024-CA-05320	Septic arthritis	Zoetis (Belgium)	Yes	Ongoing	Robenacoxib
9 Dec 2024	USA-ZOETISPV-2024-US-49215	Collapse of leg	Zoetis (Belgium)	Yes	Ongoing	No

Date	Reference	Reaction	Sender	Serious	Outcome	Other medications
9 Dec 2024	USA-ZOETISPV-2024-US-49301	Joint swelling, abnormal radiograph finding	Zoetis (Belgium)	No	Ongoing	Carprofen
9 Dec 2024	USA-ZOETISPV-2024-US-49547	Swollen joint	Zoetis (Belgium)	No	Unknown	No
10 Dec 2024	CAN-ZOETISPV-2024-CA-05327	Joint swelling, abnormal radiograph finding	Zoetis (Belgium)	Yes	Ongoing	Meloxicam
10 Dec 2024	GBR-ZOETISPV-2024-UK-05354	Arthritis, abnormal radiograph and cytology	Zoetis (Belgium)	Yes	Unknown	Grapiprant
10 Dec 2024	GBR-ZOETISPV-2024-UK-05363	Joint inflammation, bone and joint disorder	Zoetis (Belgium)	No	Unknown	No
10 Dec 2024	USA-ZOETISPV-2024-US-49657	Luxation/subluxation, plantigrade posture	Zoetis (Belgium)	No	Ongoing	No
10 Dec 2024	USA-ZOETISPV-2024-US-49914	Collapse of leg	Zoetis (Belgium)	No	Ongoing	No
11 Dec 2024	USA-ZOETISPV-2024-US-43676	Luxation/subluxation, synovitis	Zoetis (Belgium)	No	Ongoing	Grapiprant
11 Dec 2024	USA-ZOETISPV-2024-US-49976	Collapse of leg	Zoetis (Belgium)	Yes	Ongoing	No
11 Dec 2024	USA-ZOETISPV-2024-US-50172	Swollen joint, lameness	Zoetis (Belgium)	No	Ongoing	Grapiprant
11 Dec 2024	USA-ZOETISPV-2024-US-50174	Arthritis, abnormal radiograph finding	Zoetis (Belgium)	No	Ongoing	No
12 Dec 2024	AUS-ZOETISPV-2024-AU-02434	Lameness, abnormal radiograph finding	Zoetis (Belgium)	Yes	Ongoing	Lokivetmab
12 Dec 2024	CAN-ZOETISPV-2024-CA-05393	Joint effusion, abnormal radiograph finding	Zoetis (Belgium)	Yes	Unknown	No
12 Dec 2024	CAN-ZOETISPV-2024-CA-05486	Luxation/subluxation, abnormal radiograph finding	Zoetis (Belgium)	Yes	Unknown	Meloxicam
12 Dec 2024	CAN-ZOETISPV-2024-CA-05489	Luxation/subluxation, abnormal radiograph finding	Zoetis (Belgium)	No	Ongoing	Carprofen
12 Dec 2024	CAN-ZOETISPV-2024-CA-05507	Luxation/subluxation, abnormal radiograph finding	Zoetis (Belgium)	Yes	Unknown	Butorphanol, ketamine
12 Dec 2024	GBR-VMDDEFRA-02421/24	Ligament disorder, abnormal radiograph finding	Boehringer Ingelheim	No	Ongoing	Firocoxib
12 Dec 2024	GBR-ZOETISPV-2024-UK-05435	Arthritis, unable to stand	Zoetis (Belgium)	Yes	Fatal	No
12 Dec 2024	USA-ZOETISPV-2024-US-38409	Luxation/subluxation, fracture	Zoetis (Belgium)	No	Ongoing	No
12 Dec 2024	USA-ZOETISPV-2024-US-50209	Swollen limb, non-weight bearing lameness	Zoetis (Belgium)	Yes	Ongoing	No
12 Dec 2024	USA-ZOETISPV-2024-US-50268	Swollen joint, abnormal radiograph finding	Zoetis (Belgium)	Yes	Ongoing	No
12 Dec 2024	USA-ZOETISPV-2024-US-50768	Chondrosarcoma	Zoetis (Belgium)	Yes	Ongoing	No
17 Dec 2024	USA-ZOETISPV-2024-US-50455	Musculoskeletal disorder, unable to stand	Zoetis (Belgium)	No	Ongoing	Carprofen
17 Dec 2024	USA-ZOETISPV-2024-US-50741	Ligament rupture	Zoetis (Belgium)	No	Ongoing	No
19 Dec 2024	AUS-ZOETISPV-2024-AU-02469	Limb non-weight-bearing	Zoetis (Belgium)	Yes	Unknown	No
19 Dec 2024	AUS-ZOETISPV-2024-AU-02471	Fracture	Zoetis (Belgium)	Yes	Ongoing	No
19 Dec 2024	AUS-ZOETISPV-2024-AU-02480	Abnormal radiograph finding	Zoetis (Belgium)	Yes	Fatal	No
19 Dec 2024	CAN-ZOETISPV-2024-CA-05512	Swollen limb, limb non-weight-bearing	Zoetis (Belgium)	Yes	Recovered/normal	No
19 Dec 2024	USA-ZOETISPV-2024-US-46310	Swollen limb, bone and joint disorder	Zoetis (Belgium)	No	Ongoing	No
23 Dec 2024	GBR-VMDDEFRA-02275/24	Arthritis, overdose	Zoetis (Belgium)	No	Ongoing	No

Date	Reference	Reaction	Sender	Serious	Outcome	Other medications
23 Dec 2024	NZL-ZOETISPV-2024-NZ-00204	Limb non-weight-bearing	Zoetis (Belgium)	Yes	Fatal	No
23 Dec 2024	USA-ZOETISPV-2024-US-51118	Arthritis, bone and joint disorder	Zoetis (Belgium)	No	Unknown	Grapiprant
23 Dec 2024	USA-ZOETISPV-2024-US-52247	Ligament disorder	Zoetis (Belgium)	No	Ongoing	Grapiprant
24 Dec 2024	SWE-DECHRALT-2024-11131	Luxation/subluxation	Dechra Ltd	No	Recovered/normal	No
25 Dec 2024	AUS-ZOETISPV-2024-AU-02549	Abnormal radiograph finding, arthritis	Zoetis (Belgium)	Yes	Ongoing	No
25 Dec 2024	CAN-ZOETISPV-2024-CA-05606	Ligament rupture, tendon injury	Zoetis (Belgium)	Yes	Unknown	Pentosan polysulphate
25 Dec 2024	DEU-ZOETISPV-2024-DE-01673	Lameness	Zoetis (Belgium)	No	Recovered/normal	No
26 Dec 2024	GBR-ZOETISPV-2024-UK-05699	Arthritis, bone and joint disorder, abnormal radiograph	Zoetis (Belgium)	Yes	Unknown	Lokivetmab, firocoxib
27 Dec 2024	DEU-DEUBVLVP-2024-04890	Joint swelling, abnormal radiograph finding	Zoetis (Belgium)	Yes	Ongoing	Firocoxib
